# Can children and adolescents with ADHD use attention to maintain verbal information in working memory?

**DOI:** 10.1371/journal.pone.0282896

**Published:** 2023-03-14

**Authors:** Luísa Superbia-Guimarães, Michel Bader, Valérie Camos

**Affiliations:** 1 Department of Psychology, University of Psychology, Fribourg, Switzerland; 2 University Service of Child and Adolescent Psychiatry, University Hospital of Lausanne, Lausanne, Vaud, Switzerland; Liverpool John Moores University, UNITED KINGDOM

## Abstract

Children and adolescents with attentional-deficit/hyperactivity disorder (ADHD) present deficits in working memory (WM), but accounts for this phenomenon are still lacking. In this study, we used two variations of a complex-span task to test whether a specific WM mechanism, attentional refreshing, causes these deficits. Attentional refreshing is a maintenance strategy based on the sequential switch of attention between maintaining and processing information in WM. Its use is evidenced by a decrease in recall performance proportional to the distraction of attention away from the memoranda. In this study, we designed two experiments requiring children and adolescents with ADHD symptoms to maintain sequences of letters for subsequent recall, while performing a distracting task. In Experiment 1, the distracting task consisted of reading digits aloud. In Experiment 2, it consisted in making spatial judgements. The pace of the distracting tasks was varied to manipulate the level of attentional distraction. We observed that recall in ADHD participants was higher in the distracting conditions that give attention more opportunity to refresh letters. Moreover, ADHD participants had a similar recall performance to their age-matched typically developing peers. This study shows first evidence that individuals with ADHD can use attention to maintain verbal information in WM and calls for more research to understand their WM development.

## Introduction

Daily life requires people to constantly plan, adapt, and execute actions according to information currently available in their minds. At a moment, one is driving home and then they remember they have to go to the grocery store, so they must change their route. Working memory (WM) is the cognitive system allowing people to flexibly maintain and process information during short delays to accomplish goal-directed behaviour–that is, to change the route halfway through it and to recall items in a shopping list, whilst planning dinner. As such, WM sustains nearly every daily-life activity including complex cognitive activities such as verbal reasoning, verbal production and comprehension, mathematical cognition, spatial navigation, among others [1–5].

Deficits in WM functioning are common to many psychopathologies, including attention-deficit/hyperactivity disorder (ADHD), a neurodevelopmental disorder affecting 7.2% of children in the world [6]. ADHD is characterized by a pervasive pattern of hyperactivity/impulsivity and/or inattentive behaviours that persists in time (at least 6 months) and is present in different life contexts [7]. There are three different possible clinical subtypes of ADHD, depending on the prevailing symptoms: predominantly hyperactive/impulsive, predominantly inattentive, and combined subtype. Individuals with all subtypes of ADHD classically present poorer performance than their typically developing peers in WM tasks. This includes the verbal and visuospatial domains, and the central executive component of WM, which is in charge of the control of cognitive processes [8]. Meta-analyses revealed that visuospatial WM and the central executive are particularly more affected by ADHD, with greater effect sizes of an ADHD diagnosis upon performance in these domains [9–11]. For example, Kasper et al. [11] reported effect sizes of 0.74 (Hedge’s g) in visuospatial WM and 0.69 in verbal WM in a meta-analysis of 45 studies. Moreover, Martinussen et al. [9] subdivided the tasks reported in the literature according to their requirements of storage and central executive processing. They found greater effect sizes upon the central executive processing component of both spatial and verbal WM, with greater impairments in the spatial domain.

The WM deficits mentioned above are detected by different measurements ranging from simple neuropsychological tests to more complex constructs such as intelligence and school achievement [12–14]. Most studies on WM functioning in ADHD are based on the neuropsychological approach, in which patients are assessed by test batteries or behavioural inventories acknowledged to directly reflect their WM capacities. Examples of test batteries and behavioural inventories are the Wechsler Intelligence Scale for Children–WISC [15], the Working Memory Test Battery for Children–WMTB-C [16], and the NEPSY-II [17]. The scores from neuropsychological tests and behavioural inventories inform us about one’s performance related to a normative group; therefore, they are helpful tools to study individual and group differences regarding the psychological constructs they were designed to measure. However useful this might be to characterize the cognitive functioning of clinical groups, the classical neuropsychological approach is not able to pinpoint specific psychological processes that underlie poorer WM performance. For instance, neuropsychological tests are insufficient to explain the particularities regarding the visual and visuospatial domains mentioned above or the involvement of the central executive component. In other words, the neuropsychological approach can reveal that individuals with ADHD perform WM tasks poorer than typically developing individuals, but it cannot provide a functional explanation for this phenomenon.

The time-based resource-sharing (TBRS) model of WM [18–20] can provide a mechanistic insight into why ADHD affects WM performance. According to the TBRS model, a core feature of WM is the ability to rapidly switch attention between processing and maintaining information to reactivate memory traces, a process called attentional refreshing. Attentional refreshing is a maintenance mechanism of WM together with phonological rehearsal, but, differently from the latter, it is a domain-general mechanism. Because attention cannot be simultaneously split between processing and maintenance demands and it must be sequentially allocated to processing and maintenance, the time course of a task will directly impact one’s capacity to perform attentional refreshing.

The TBRS model has specific predictions on how the time course of a task affects attentional refreshing and thus memory performance. The ratio between the time of the attentional capture (*Ta*) and the total time available to process stimuli (*Tt*) during a task is conceptualized by the TBRS model as the cognitive load (CL) of this task, that is, CL = *Ta*/*Tt*. The CL of a task will directly impact WM performance, with higher CLs causing participants to recall fewer items and vice-versa. Simply put, the less time one has to switch attention back and forth from concurrent processing and maintaining information in WM, the poorer their memory performance will be.

Given their paper-and-pencil format and the lack of refined control of temporal parameters, the neuropsychological testing approach is not sensible to the CL effect, nor can it detect the use of attentional refreshing during WM tasks. The experimental research approach offers a reliable alternative to assess it. Researchers can manipulate the cognitive load of a WM task by using a complex span paradigm, in which increasingly longer sequences of memory items (e.g., letters to memorize) are interspaced with a concurrent processing task (e.g., to read digits aloud) [21]. At the end of each trial, the participant is required to recall the presented sequence, and the length of the correctly recalled sequences is a dependent variable used as an estimate of one’s WM capacity (i.e., their memory span). In the complex span paradigm, CL is manipulated by varying the pace of presentation of stimuli of the processing task during the interval (*Tt*) in-between each memory item of the sequence. When the processing task is performed at a fast pace, CL is higher, thus causing a drop in WM performance. Conversely, when the processing task is performed at a slow pace, the CL is lower and WM performance is improved.

The effect of the CL on WM performance has been experimentally observed in different age groups within the typically developing population [18, 21–24], but it has never been tested in clinical populations. According to the TBRS model, attention is needed to maintain information in the short term. Because children with ADHD symptoms present specific disturbances of attention, the present study tested, first, whether they employ attention to refresh information in WM. If so, the CL of the concurrent task should impact their memory performance in a complex span task. Alternatively, their WM performance should be immune to any CL effect if they do not employ attention to maintain items in WM. Second, if children with ADHD symptoms employ attention to maintain items in WM, but less efficiently, the impact of the CL on their WM performance should be weaker than what is commonly reported in the literature in typically developing children (e.g., [21]). Finally, we aimed to generalize the use of the complex span paradigm to assess the CL effect in children with ADHD. In doing so, we hope to provide an alternative method to study WM processes in populations with ADHD.

## Study rationale and hypothesis

The present study implemented two complex span tasks manipulating the CL. We ran two experiments in parallel to test our hypotheses relating the CL of the task and the recall performance in children with symptoms of ADHD. The experiments were conducted in parallel for the sake of coping with time limitations and restrictions in data collection caused by the COVID-19 pandemic. Experiment 1 implemented the reading-digit span task of Barrouillet et al. [21] in which the pace of presentation of the stimuli in the processing component was varied to manipulate the CL of the task. In this task, children are asked to memorize sequences of letters visually presented on the screen and interspaced with visually presented digits. They are required to read aloud all the stimuli presented on the screen, but only to recall the letters. By varying the pace of presentation of the digits between each letter (from the highest to the lowest CL: 2 digits/second, 1.2 digit/second, 0.8 digit/second, and 0.4 digit/second), Barrouillet et al. [21] found a significant effect of the CL in typically developing children aged 8, 10, 12, and 14 years, with lower CLs yielding higher mean spans, as predicted by the TBRS model. Moreover, the effect of the CL on the mean span was different across age groups, with steeper slopes for the older children. This slope is taken as an index of the efficiency of attentional refreshing, with steeper slopes indicating that children benefited more from the free time in low CL conditions to refresh items in WM.

In Experiment 2, we tailored the pace of the processing task according to each participant’s mean reaction time (RT) to equalize the CL across participants and groups. We had three general hypotheses. First, the CL effect should be observed in the ADHD group in Experiment 1 if this population uses attentional refreshing to maintain items in WM. Second, the ADHD group should exhibit a less efficient attentional refreshing if it uses this mechanism. Third, the equalization of the CL in Experiment 2 should abolish any purported group differences attributed to a failure or a weaker efficiency in using refreshing between ADHD and controls. We will detail the specific rationale and predictions of Experiments 1 and 2 in the following sections.

## Materials and method

### General method

#### Recruitment of participants

Fifteen participants aged between 10 and 16 (Mean age = 13.17 years, *SD* = 1.7 years, four girls) with a pre-existing diagnosis of ADHD and 36 typically developing controls (mean age = 12.6 years, *SD* = 2.2, forty girls) took part in the study. The endpoint of our recruitment was determined by COVID-19 restrictions. Children in the ADHD group were recruited via the “Association Suisse Romande de Parents d’Enfants avec Déficit d’Attention, avec ou sans Hyperactivité” (ASPEDAH) and the second author’s private medical clinic. Children in the control group were recruited in public schools in the canton of Fribourg. All parents gave written consent for their child’s participation in the study, and adolescents older than 14 were also required to sign the consent statement, following local ethical guidelines. The ADHD group included participants previously diagnosed with all clinical subtypes of ADHD (predominantly inattentive, predominantly hyperactive-impulsive, and combined subtype). The absence of comorbid neurodevelopmental disorders and executive functioning deficits were exclusion criteria for participation in the study. The presence of ADHD symptoms and the absence of comorbid disorders were assessed by a set of questionnaires and scales previously filled in by parents or legal guardians during the recruiting phase of the study. This set of questionnaires and scales included the Child Behavior Checklist (CBCL, [25]); the Conners-3 [26], the ADHD rating scale (ADHD-RS, [27, 28]), the Behavioral Rating Inventory for Executive Function (BRIEF, [29]), and a questionnaire on the child’s medical history and medication intake for children in the ADHD group. Parents in the control group were required to fill in only the CBCL and the Conners-3. This study received ethical agreement from the local authority in Switzerland (Commission cantonale d’Ethique de la Recherche sur l’être humain, CER-VD, agreement number 2021–01087).

#### Apparatus

The experiments were programmed and implemented using the software PsychoPy v.2020.1.3 [30] on a laptop computer (HP Probook 440 G6). The computer’s screen measured 14 inches with a resolution of 1920 x 1080 pixels, and the refreshing rate was 60 Hz.

#### Experimental sessions

The experimental sessions were carried out individually and on separate days. Participants under psychostimulant medication in the ADHD group were asked to cease the intake at least 24 hours prior to the experimental sessions. Most sessions with the ADHD participants were scheduled on weekends and/or school holidays to minimize the disturbance on the family’s routine, since it is usual that children and adolescents with ADHD take the medication only on school days. The experimental sessions were carried out in a silent room in a place chosen according to the family’s convenience (e.g., the family’s house, a public library, the University of Fribourg). Testing ADHD participants outside school avoided disturbances in the school environment due to the cessation of medication intake and missing class time. In the control group, sessions were scheduled during school hours in a separate room in the school. The computer was placed about 60 centimetres of distance in front of the participant.

### Experiment 1

In Experiment 1, we implemented Barrouillet et al.’s [21] reading digit span task with the same method to manipulate the CL of the task and to test whether variations in the CL affect memory performance in children with ADHD. In particular, we manipulated CL by keeping constant the interval between each memory letter (*Tt* = 10 s) and varying the number of digits presented during this interval to create two pace conditions: a fast (1.2 digit/second) and a slow (0.4 digit/second) pace. Because children with ADHD have difficulties staying focused during long periods, we contrasted only two pace conditions from Barrouillet et al. [21] instead of using four pace conditions as they did in the original study. As explained in the introduction, the slow pace yields a lower CL because participants have more spare time to switch attention away from processing the digits (i.e., to read them aloud) to maintain the sequence of letters. Conversely, the fast pace yields a high CL. If children and adolescents with ADHD, despite their deficit, use attention to refresh memory traces in WM, we predicted that they should have higher mean spans in the slow pace (low CL) than in the fast pace (high CL) condition, as observed in the typically developing population. If not, their mean spans should not significantly differ between pace conditions. Second, if children and adolescents with ADHD use attentional refreshing less efficiently than typically developing individuals, then the slope relating their mean spans to the CL should be less steep than the slope of typically developing controls. According to this hypothesis, we predicted a two-way interaction between the factors group and pace condition in Experiment 1.

### Participants

Fifteen participants took part in Experiment 1 in the ADHD group, and 19 participants took part in the control group. One control was excluded from the data analysis because he was an outlier in all the dependent variables we analysed. We tested the difference between ADHD and controls in the *T*-scores of inattention and hyperactivity and, we found very strong evidence of a group difference, confirming that clinical symptoms were present in participants diagnosed with ADHD and absent in controls. The supplementary materials contain the complete characterization of participants in Experiment 1 and the complete analysis of the symptom scores between groups.

### Material and stimuli

The complex span task included consonants to be recalled and digits to be read. The stimuli pool consisted of 46 pseudo-random lists of consonants from the French alphabet, excluding the letters “W” and “Y” because they are multisyllabic in French. We avoided French acronyms in the lists and there was no repetition of letters within a list. The lists varied in length from two to eight consonants, with three lists of each length per pace condition. Two additional lists of two consonants were created for practice in each pace condition. For the secondary task of the complex span task, we created a set of 117 pseudo-random series of 12 digits and a set of 117 pseudo-random series of four digits (Hindu-Arabic numerals). We avoided obvious numerical sequences and repetitions of digits within a series (e.g., “12345”, “7777”).

The lists of letters were displayed in font “Arial” (height set to 0.1 in Psychopy settings) and in dark red (colour name “maroon”, RGB values: 128, 0, 0). The digits were displayed in the same font and size, but in white. The central fixation point was a white asterisk (font “Arial”, height set to 0.1 in PsychoPy settings). The background colour of the program window was set to grey (RGB values: 175, 175, 175).

### Procedure

Participants were presented with the same lists of two to eight consonants, in increasing length. The order of presentation of the lists was the same for all participants. Each trial began with the display of the fixation point for 750 ms, followed by 500 ms of a blank screen and the display of one letter at the centre of the screen for 1500 ms. The presentation of the letters was interspaced by an interval of 10 seconds, during which a series of digits was successively displayed at the centre of the screen. After the presentation of the last letter of the list, the word “Rappel” (“recall” in French), was displayed at the centre of the screen and the participant was asked to orally recall the sequence of letters seen in the trial, in the correct serial order.

**Fig 1:**
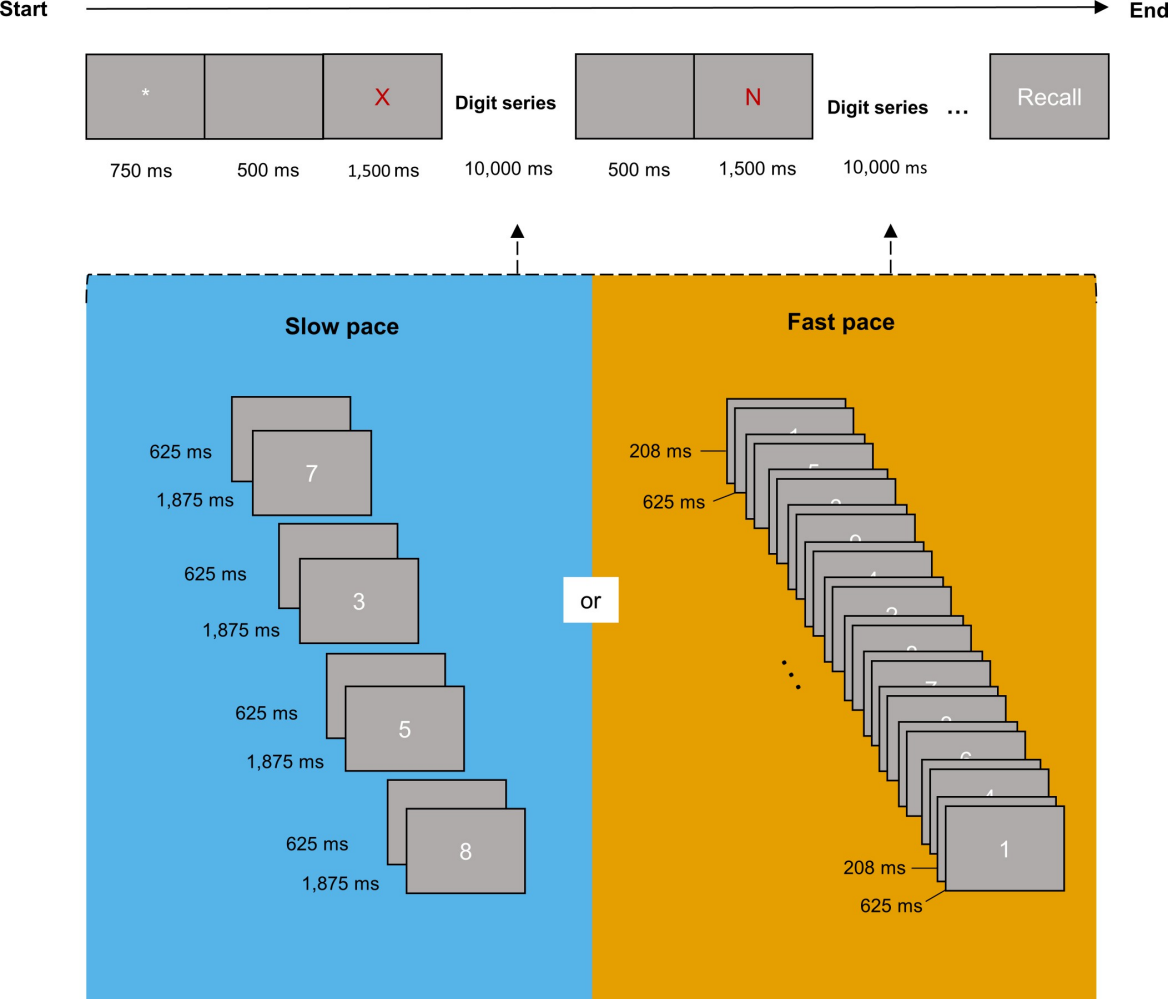
The reading-digit span task. In the task, participants were presented with lists of letters of increasing length and series of digits were interspaced between each letter in a list. To vary the cognitive load of the task, the interval between each letter was kept constant (10 s) but the number of digits presented in this interval varied (0.4 digit/second in the slow pace and 1.2 digit/s in the fast pace).

In the slow pace condition, four digits were displayed during the 10-second interval in-between letters. Each digit was displayed for 1875 ms and interspaced by 625 ms of a blank screen, totalling four periods of 2500 ms with 75% of display and 25% of inter-digit delay. In the fast pace condition, 12 digits were displayed during the 10-second interval in-between letters. Each digit was displayed for 625 ms and interspaced by 208 ms of a blank screen, totalling four periods of 833 ms with 75% of display and 25% of inter-digit delay. Hence, the slow pace condition corresponded to a ratio of 0.4 digit-per-second, and the fast-pace condition corresponded to a ratio of 1.2 digit-per-second. Participants were required to read aloud all the letters and digits presented in a trial, and to maintain the letters while keeping the pace of reading (Fig 1). To prevent the use of phonological rehearsal between digits, participants were instructed to utter each digit as long as it was displayed on the screen.

We manipulated the pace conditions between blocks of trials, and the order of presentation of the blocks was counterbalanced between participants. Each block began with two practice trials containing only digits so that participants could practice the pace of reading. Participants were instructed to read the digits aloud as they appeared on the screen and to keep the pace of uttering during the whole task. After these two practice trials, the experimenter informed the participants that they must memorize a letter appearing before the digits, and an example trial with a single letter was given. The question “Quelle était la lettre?” (“What was the letter?”) appeared on the screen and was read aloud by the experimenter.

After the participant’s oral response, the message “Mais c’est trop facile avec une seule lettre! On essaye plus dur ?” (“It’s too easy with a single letter! Let’s try harder?”) appeared on the screen and was read by the experimenter. The participant then performed two practice trials with sequences of two letters. The participant was informed that the task was challenging and that the number of letters in a sequence would progressively increase. Each block contained a maximum number of twenty-one trials, with three trials per sequence length (e.g., three trials of sequences of two letters, three trials of sequences of three letters, and so on). The length of the letter sequences increased progressively at every three trials. The word “Rappel” (“Recall” in French) appeared on the screen at the end of each trial and the participant was required to orally recall the letters in the order they had been presented. The participants’ responses were recorded by hand by the experimenter and no feedback was given during the task. The procedure ended when the participant failed to recall all three lists of letters at any given length.

### Data analysis

Our dependent variables were the mean span and the percentage of correctly recalled letters. For the sake of brevity, and because the results were congruent, we present here only the analysis of the mean span. The analysis of the percentage of correctly recalled letters were reported in the supplementary material. We ran Bayesian repeated-measures ANOVAs with JASP (v. 0.16.4.0) [31] to compare the performance between groups and pace conditions for each dependent variable. We used a 2x2 design with the group (ADHD vs. control) as a between-participant factor and the pace (fast vs. slow) as a within-participant factor. We set the prior odds for each model as equivalent (i.e., P_(M)_ = 0.2), because we found no data reports in the literature using Bayesian statistics for our experimental design and clinical population. We opted for the Bayesian analysis because it is more robust in cases of small sample sizes. Also, it allows a comparison between two predictive models rather than an estimate of the error probability in hypothesis testing, as in the frequentist approach. In the Bayesian approach, the statistical value of interest is called Bayes factor (BF_10_) and it represents a ratio between the likelihood of finding the observed data if the alternative hypothesis is true and the likelihood of finding the data if the null hypothesis is true. Thus, it represents a ratio between two conditional probabilities. A Bayes factor close to one means that the data are equally likely to be observed under the two hypotheses at stake–that is, a BF = 1 represents an outcome at the chance level. A Bayes factor close to zero means that the observed data are more likely to be found in the population if the null hypothesis is true. Conversely, a Bayes factor greater than one means that the data are more likely under the alternative hypothesis. In Bayesian ANOVAs, the Bayes factors of different explanatory models for the data are calculated. For instance, in our 2x2 design, the output Bayes factors show a comparison between the following models to the null hypothesis: (1) the main effect of the pace, (2) the main effect of the group, (3) the independent effects of the pace and the group, (4) the interaction between the pace and the group. By evaluating the magnitude of each Bayes factor, one can decide on rejecting or accepting the alternative hypothesis under investigation.

### Results

Before performing analysis on recall performance, we checked that participants were correctly performing the secondary task by computing the number of reading digit mistakes they made throughout the experiment. A mistake was counted whenever the participant did not read a digit aloud or misread the presented digit. As can be expected, the percentage of reading mistakes was very small, less than 5% of the digits in both pace conditions.

#### Mean spans

To calculate the span of each participant, we summed 1/3 to each correctly recalled list of letters and added one [21]. For example, someone who correctly recalled three lists of two, two lists of three, three lists of four, and one list of five has a span of ((3+2+3+1) * 1/3) + 1 = 4. The addition of one to the total number of thirds takes into account that our procedure started with lists of two lists, the success in recalling only one letter being assumed.

**Fig 2:**
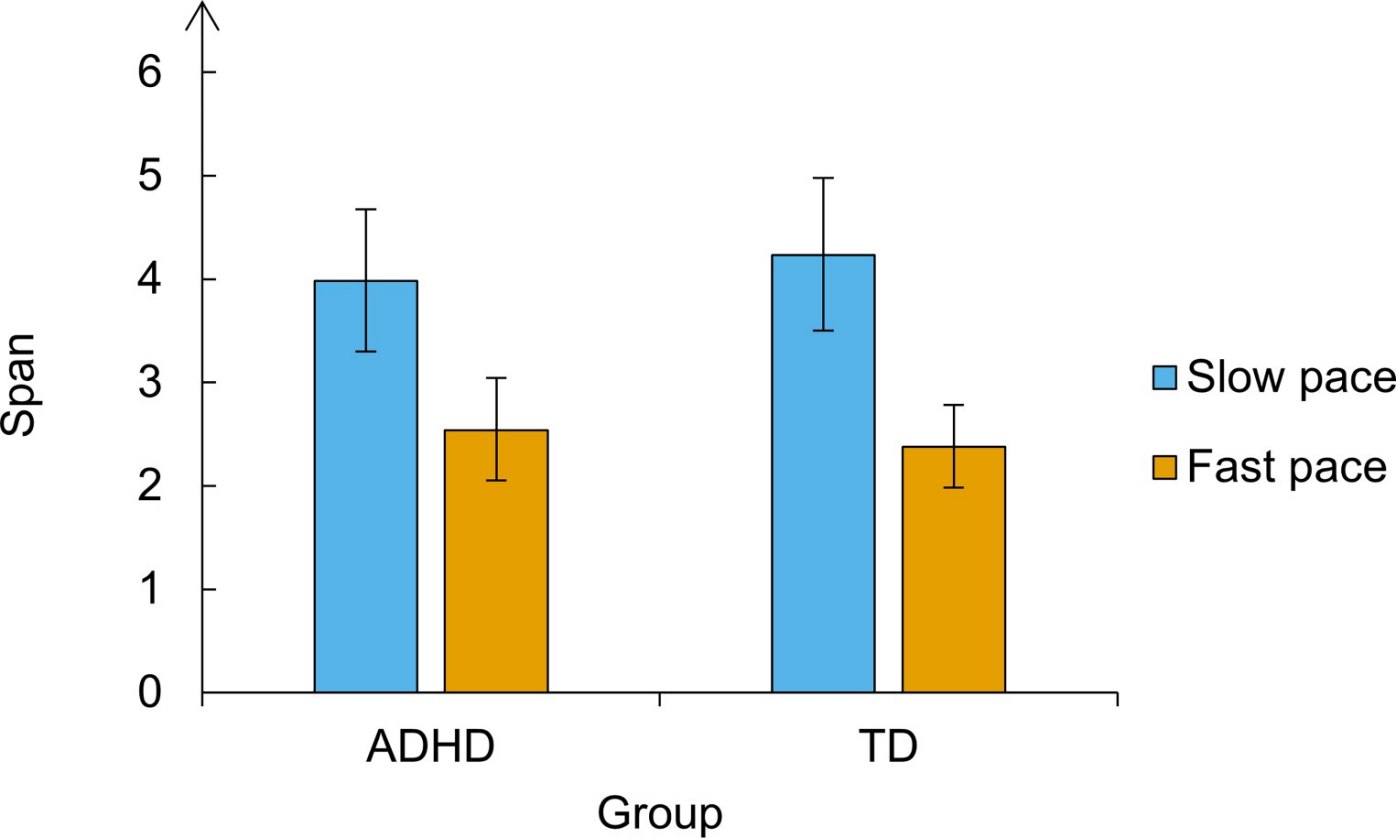
Mean spans per group and pace condition in Experiment 1. The vertical lines represent the confidence intervals.

The average mean span in the ADHD group was 3.98 (*SD* = 1.24) in the slow pace and 2.54 (*SD* = 0.89) in the fast pace. In the control group, the average mean span was 4.23 (*SD* = 1.48) in the slow pace condition and 2.38 (*SD* = 0.80) in the fast pace condition. Fig 2 depicts these results. We compared the mean spans between groups and experimental conditions by running a Bayesian repeated-measures ANOVA. According to this analysis, the model that best fitted the data was the one considering only the main effect of the pace (BF_10_ = 1.902 x 10^7^, error = 6.59). According to this model, recall performance was hampered in the fast pace, both in the ADHD and control groups. The second-best model was the additive model including the main effects of the pace and the group (BF_10_ = 0.656 x 10^7^, error = 1.04), followed by the full model including the two main effects and the interaction between them (BF_10_ = 3.372 x 10^6^, error = 3.82).

In accordance with our explanation of the Bayes analysis, the BF_10_ of 1.902 x 10^7^ of the best model means that the observed data are 1.902 x 10^7^ more likely to be observed under the alternative hypothesis than under the null. This yields very strong evidence in favour of the hypothesis that participants have higher mean spans in the fast condition. Because the Bayes factors of the two best models did not differ greatly, we examined the BF_incl_ and BF_excl_ of each factor in the models to better understand how they accounted for the data. The BF_incl_ and BF_excl_ represent the evidence for including or excluding a given factor in the model, respectively. We found extreme evidence for the inclusion of the pace effect (BF_incl_ = 1.488 x 10^7^) in the model, but not for the exclusion of the group effect (BF_excl_ = 2.76) and the interaction between group and pace (BF_excl_ = 1.88).

**Fig 3:**
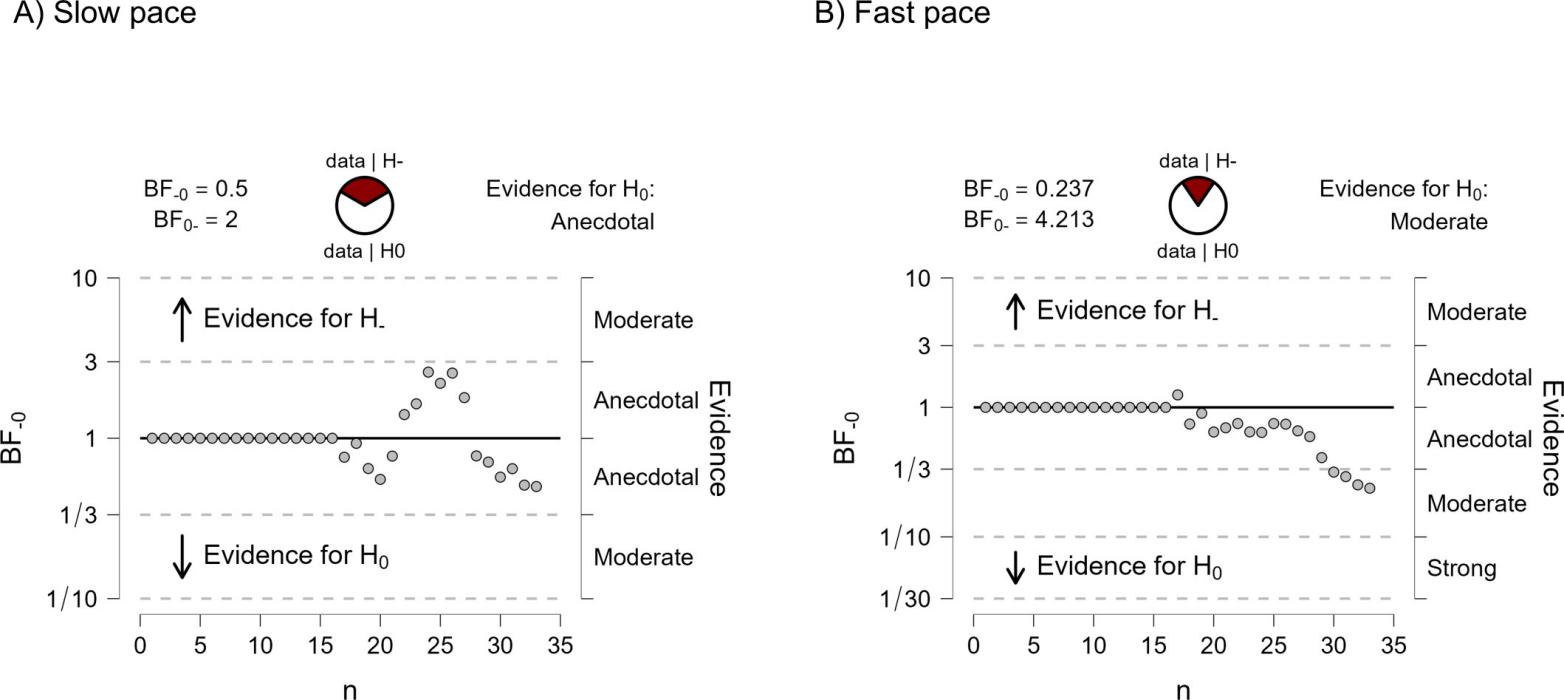
Sequential Bayesian analysis of the mean spans in Experiment 1. The panels reproduce the output from Jasp (Jasp Team, 2022) The x-axis of each plot represents the participant number and the y-axis represents the magnitude of the BF. Each point in the plot represents a change in the BF caused by the addition of a participant in the analysis. The horizontally aligned dots until n = 15, on the left side of each plot, correspond to data in the ADHD group.

The results of the Bayesian ANOVA showed a strong effect of the pace upon the mean spans of both ADHD and controls and did not confirm our hypothesis predicting an interaction between group and pace. As observed in Fig 2, the data pattern was essentially the same for ADHD and controls, despite the non-decisive evidence for the exclusion of the group effect and the interaction between group and pace in the model. To better account for a possible group effect, we ran pairwise sequential comparisons between the two groups in each pace condition using *T*-tests for independent samples. This type of analysis allowed us to visualize a trend, if any, in the cumulative evidence in favour of the null hypothesis predicting no differences between the two groups. Again, we found no evidence of a group difference between ADHD and controls in both pace conditions. Fig 3A and 3B show cumulative the trend in the Bayes factor towards the null hypothesis.

**Fig 4:**
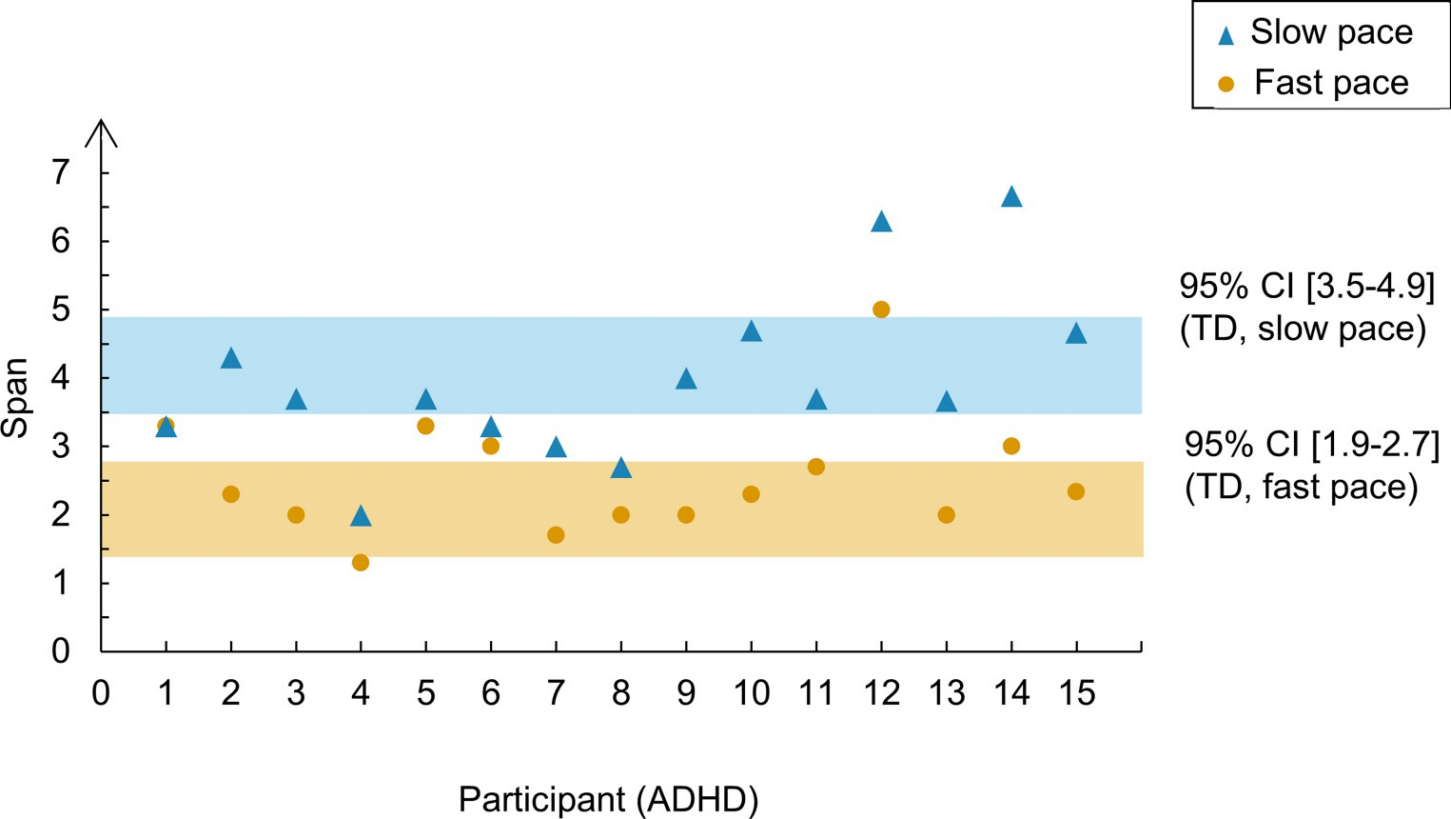
Individual spans in the ADHD group per pace condition in Experiment 1. Each data point in the graph represents an individual span in the ADHD group. The shaded zones represent the 95% confidence intervals of the mean in the control group.

The results presented so far support the interpretation that the fast pace disrupted performance in both groups and that ADHD participants were no less affected by the increased pace than the typically developing participants. Because our sample of ADHD children was heterogeneous regarding the age of participants and their *T*-scores of inattention and hyperactivity, we hypothesized that these variables can modulate the observed CL effect. Fig 4 shows the individual spans of ADHD participants per pace condition plotted against the 95% confidence intervals of participants in the control group. It allows us to visualize the effect of the fast pace upon each participant’s span. Some participants in the ADHD group were greatly affected by the fast pace (e.g., participant 14), whereas others were just mildly affected by it (e.g., participants 5 and 6).

It is possible that participants with higher levels of inattention were less affected by the fast pace because their baseline performance in the slow pace was already poor, suggesting a difficulty in implementing attentional refreshing even when the processing component of the task allows plenty of free time to do it. Moreover, there is a developmental trend in the use of attentional refreshing, with children becoming more proficient at the beginning of adolescence [21, 32]. As children reach adolescence and implement refreshing more efficiently, they become more prone to pace manipulations, i.e., their performance is more hampered by high CL compared to younger children.

We ran a Bayesian repeated-measures ANOVA with the factors age, inattention, and hyperactivity as covariates to examine the impact of these possible variables on recall performance. Both ADHD and controls were included in this analysis. The model that best fitted the data was the one including the main effects of pace and age (BF_10_ = 1.603 x 10^8^, error = 1.162). The second-best model was the one including the main effects of pace, age, and inattention, with a Bayes factor very close to the best model (BF_10_ = 1.6 x 10^8^, error = 1.43). We found decisive evidence for the inclusion of the pace effect (BF_incl_ = 4.39 x 10^7^) and moderate evidence for the inclusion of the age effect (BF_incl_ = 9.13) in the model. Regarding the *T*-scores of inattention, the evidence in favour of their inclusion in the model was anecdotal (BF_incl_ = 1.293). According to these results, older participants were more affected by the fast pace than younger participants, as reported in the literature for typically developing children and adolescents [21]. Moreover, although the second-best model suggests that there was a small modulation of the inattentive symptoms, the influence of age outweighed the influence of inattention upon recall performance. To summarize, we observed a strong effect of the pace manipulation upon the mean spans in Experiment 1 and no group differences between ADHD and typically developing participants. Finally, the analysis including age and inattention scores as covariates showed that the age of participants accounted for most of the differences in their spans, in agreement with previous results in the literature [21, 33, 34].

Lastly, to account for possible confounding effects related to the order of presentation of the blocks, we ran repeated-measures Bayesian ANOVAs with the pace condition as a within-subject factor (slow pace x fast pace) and the order of presentation as a between-subject factor (fast-slow x slow-fast). We found no evidence of the effect of the order of the blocks upon the mean spans, with all BF_10_ for the models predicting an order effect or an interaction between pace and order falling in the range between 0.0 and 0.5.

### Discussion

Experiment 1 was designed to investigate if children with ADHD use attentional refreshing to maintain items in WM, and if they do so, whether their use of refreshing is less efficient than for the control group. To this end, we varied the pace of presentation of the processing component of a complex span task to induce an increase in the CL. As explained, the CL effect indexes one’s ability to use free time to employ attention to refresh items, with higher CLs being more deleterious to the use of refreshing. We expected typically developing controls to be proficient in using attentional refreshing and to be affected by the manipulation of the pace–as we indeed observed in the results. Children with ADHD, on the other hand, have pervasive symptoms of inattention and/or hyperactivity that linger throughout their development. For this reason, we did not expect them to use attentional refreshing as efficiently as the control group.

Our hypothesis was contradicted by the results of Experiment 1, in which we observed a very strong effect of the pace manipulation upon the mean span of both ADHD and controls. The deleterious effect of the fast pace upon performance reveals the CL effect predicted by the TBRS model in our sample. Specifically, the presence of a pace effect in the ADHD group suggests that the WM functioning of this clinical population is subject to the same temporal constraints as typically developing individuals [22, 35, 36]. Moreover, the presence of the CL effect in children and adolescents with ADHD suggests that this population is able to employ attentional refreshing to maintain active representations in WM. To our knowledge, our Experiment 1 is the first replication of the CL effect in a sample of ADHD participants during a verbal serial recall task.

A study by Weigard and Huang-Pollock [37] has previously shown that a manipulation of the CL affects performance of ADHD children (ages 8–10 years) in a complex span task tapping the visuospatial domain of WM. The task used by these authors consisted in memorizing spatial sequences (i.e., squares in a spatial array) and making numerosity discriminations between each item of the sequence. To manipulate the CL of the task, the authors induced slower processing speeds in the numerosity discrimination task by varying its difficulty, with more difficult judgements inducing slower processing times. They set a condition in which numerosity judgements were hard to make and another in which they were easy. The hard condition induced slower processing times and accounted for a longer decision-making process, which in turn induced an increase in the CL of the task. The results showed that the higher CLs hampered recall of the spatial sequences and, more importantly, the causal relationship between slower processing speed, CL, and recall was observed in both ADHD and typically developing controls. Moreover, the authors also did not find an interaction between the speed condition (slower vs. fast processing speed) and the group. This result is consistent with our observations in Experiment 1, in which we did not find the hypothesized two-way interaction between pace and group. The absence of such interactions both in our Experiment 1 and in Weigard and Huang-Pollock’s study [37] suggests that the WM performance of children with ADHD is similarly affected by changes in the CL. Taken together, those results provide first evidence that children with ADHD are prone to temporal constraints affecting the use of attentional refreshing to maintain items in WM.

In typically developing children, the use of attentional refreshing emerges by the age of 7 and achieves adult levels of efficiency by the age of 14 [18, 21]. In our results, the inclusion of the factor age as a covariate in the ANOVA suggests that the ability to refresh items in WM follows the same developmental trend as in typically developing children. For a similar age group to ours (i.e., 12-year-olds) and using the same task, Barrouillet et al. [21] reported a mean span of around 5 letters in the slow pace (0.4 digit/s) and 3.3 letters in the fast pace (1.2 digit/s). In our Experiment 1, typically developing controls had a mean span of 4.2 in the slow pace and 2.3 letters in the fast pace. ADHD participants, in their turn, had a mean span of 3.9 in the slow pace and 2.5 letters in the fast pace. Participants in the study by Barrouillet et al. [21] outperformed both our ADHD and control participants in the reading digit span task in about one letter, but the drop in performance caused by the fast pace was similar across participants in the two studies. It caused a drop of about 1.7 letters in typically developing children tested by Barrouillet et al. [21], 1.9 in our typically developing controls, and 1.4 in our ADHD participants. Although direct comparisons between two studies should be done carefully, this comparison suggests that, at least in our sample, ADHD children were subject to a CL effect of similar magnitude than previously reported for the same age group of typically developing individuals.

### Experiment 2

We designed a second experiment to test another prediction of the TBRS model relating the CL and recall memory in children with ADHD symptoms. The TBRS model predicts that recall performance is inversely proportional to the CL of a task. Therefore, two predictions derive from it. First, pace manipulations enabling more free time to use refreshing will alleviate the CL and consequently cause an improvement in recall memory. Second, the equalization of the CL across participants should alleviate individual differences in performance and enable similar mean spans [22]. Experiment 2 was designed to test these two predictions in children with ADHD.

Experiment 2 has two fundamental differences from Experiment 1. First, we used a spatial judgement task (henceforth “spatial fit”) instead of the reading digit task as a concurrent processing task between the memory items (i.e., the letters). Contrary to the reading digit task that disrupts the use of articulatory rehearsal, the spatial fit mainly disrupts the use of attention refreshing. Second, we manipulated the pace of the concurrent processing task in one block of trials by adapting it to each participant’s mean RT in the spatial fit task. Therefore, participants carried out the task at a predetermined pace (baseline condition) and at an individualized pace. The implementation of a mouse response to the spatial fit allowed us to calculate each participant’s RT and use it to individualize the pace of the processing concurrent task to their own processing speed. The adaptation of the pace should attenuate individual differences in processing speed and thus equalizes the CL between participants and groups.

As explained in the introduction, the TBRS model conceptualizes the CL as the ratio between the time that attention is occupied by a concurrent processing task (*Ta*) and the total time available to perform it (*Tt*), that is, CL = *Ta/Tt*. Any individual difference in participants’ processing speed will affect the Ta and the CL of the task, e.g., faster participants have a lower *Ta* (because they rapidly free attention from processing demands) and thus the CL is also lower. In sum, providing participants with the same pace of presentation of stimuli (as we did in Experiment 1)–does not warrant the same CL across participants. To control for differences in the CL, we must adapt the *Tt* according to each participant’s individual *Ta*.

Experiment 2 was therefore a replication of Experiment 1 with an extra control variable, the CL. In the baseline condition, the interval to perform the spatial fit was pre-determined, which implies that any individual differences in RTs between the ADHD group and controls yield different CLs between the groups and can cause differences in recall performance. In the adapted condition, we adjusted *Tt* according to each participant’s mean RT in the spatial fit task, thus equalizing the CL between participants and groups. Therefore, Experiment 2 had a control baseline condition in which the CL was different between groups and an adapted condition in which the CL was the same between groups. Table 1 presents a summary of the differences and similarities between Experiments 1 and 2.

**Table 1:** Summary of the experimental design of Experiment 1 and Experiment 2.

	Experiment 1 The reading digit span task	Experiment 2 The adapted span task
Memory task	To recall lists of letters of increasing length (2 to 8 letters).	
Processing task	To read digits aloud.	To make spatial judgements.
Response modality	Serial recall (oral responses) and reading aloud the digits.	Serial recall (oral responses) and mouse clicks for the spatial fit task.
Pace conditions	Slow pace (0.4 digits/s)	Baseline pace (predetermined, 1,500 ms per spatial judgement)
	Fast pace (1.2 digits/s)	Individualized pace (1.5 x mean RT per spatial judgement)
CL conditions	Lower CL (slow pace)	Variable CL per participant (baseline pace).
		CL = RT/1,500ms.
		CL _(ADHD)_ ≠ CL _(control)_
	Higher CL (fast pace)	Same CL per participant (individualized pace)
		CL = RT/1.5x RT)
		CL _(ADHD)_ = CL _(control)_
Predictions	If ADHD children can perform attentional refreshing, memory performance should be higher in the low CL condition in both groups.	If ADHD deficit in WM results from slower attentional refreshing, their memory performance should be closer to controls when the same CL is warranted.

Gaillard et al. [22] already used an experimental manipulation adapting the pace of the task to equalize the CL between groups of participants. They showed that the adaptation of the pace according to each participant’s processing speed abolished age differences between typically developing 8- and 11-year-olds, in accordance with the predictions of the TBRS model. In our study, we predicted that such equalization would reduce any purported differences in performance between ADHD and control groups attributed to misuse of attentional refreshing and would also reduce the variability in recall performance observed in Experiment 1. As mentioned previously, we ran experiments 1 and 2 in parallel due to time constraints in data collection due to the COVID-19 pandemic, therefore they were both designed under the assumption of a deficit in using attentional refreshing by ADHD children. It is well documented in the literature that children with ADHD have slower processing speed and more variable RTs than their typically developing peers [38–41]. For this reason, we hypothesized that the CL would be different between ADHD and controls in the baseline condition (pre-determined pace), causing group differences in performance. On the other hand, the CL should be equalized between groups in the individualized condition, erasing group differences between ADHD and controls.

### Participants

Fourteen participants took part in the ADHD group (3 females, mean age = 13.7 years, *SD* = 1.7) and 17 participants took part in the control group (9 females, mean age = 12.2 years, *SD* = 1.4). All participants in the ADHD group and one participant in the control group had previously taken part in Experiment 1. We excluded two outliers in the first percentile of data in the control group, so that the final data analyses included 15 participants (7 females, mean age = 12.3 years, *SD* = 1.4). The complete sample characterization and the analysis of group differences in the *T*-scores of inattention and hyperactivity can be found in the supplementary material.

### Materials and stimuli

The same stimuli pool of Experiment 1 was used for the letter sequences. For the spatial fit task, we created 96 images of a horizontal bar sided by two squares (sides 0.5 cm) horizontally aligned. The width of the horizontal bar was 0.5 cm, and its length ranged from 1.1 cm to 5.2 cm. The gap between the two squares varied from 0.5 to 5.4 cm. The horizontal bar was located above or below the two squares, and the vertical distance between the bar and the squares varied from 0.5 to 1.7 cm. The horizontal bar fitted the gap between the two squares in half of the stimuli. A Bluetooth mouse device was connected to the laptop to enable response collection in the spatial fit task.

### Procedure

**Fig 5:**
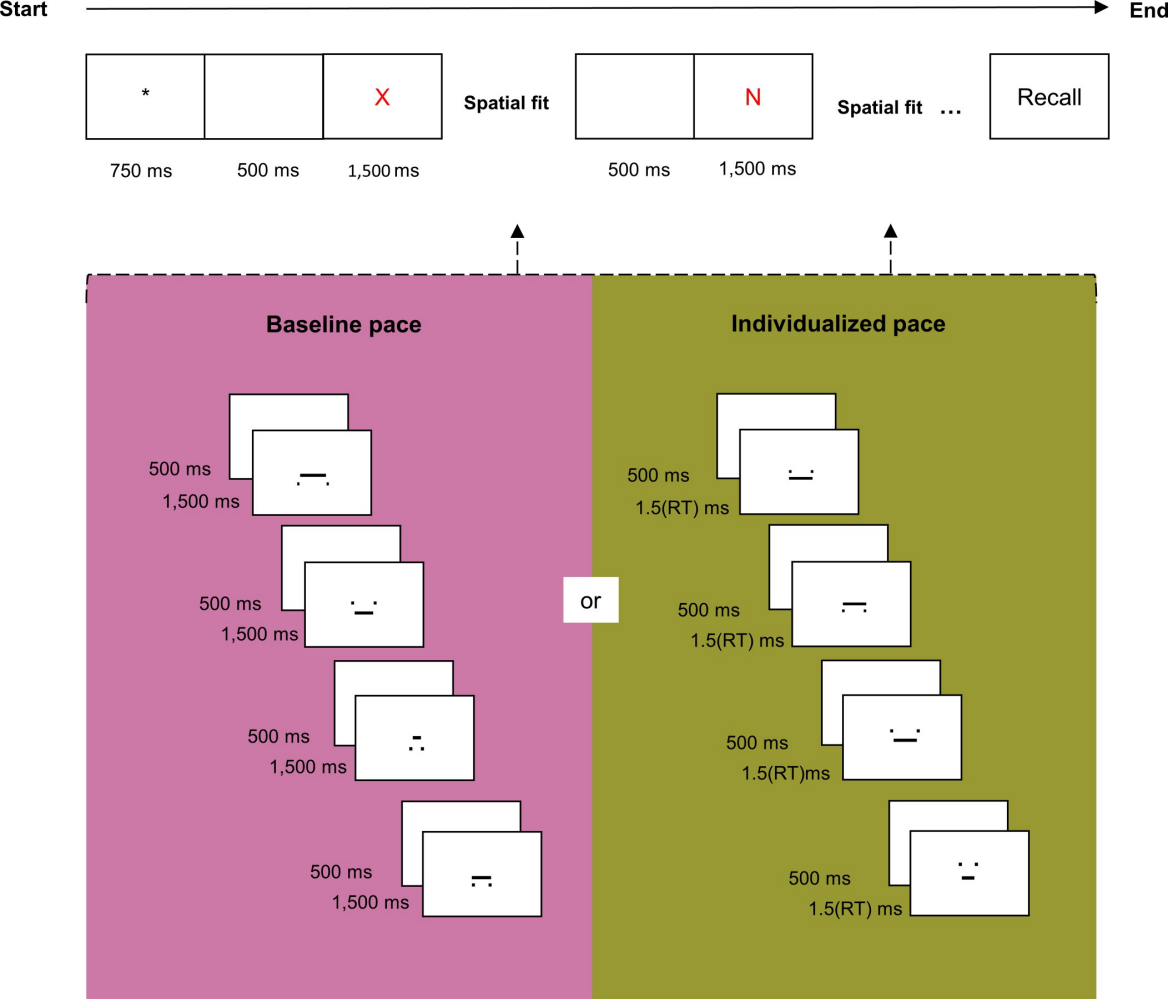
Procedure of Experiment 2. Participants were presented with increasingly longer lists of letters and the letters were interspaced with a spatial fit task. Participants were required to respond if the horizontal bar fit the gap between the two squared. The duration of the fit space task was the same for all participants in the baseline condition and it was adapted according to each participant’s mean RT in the individualized condition.

Experiment 2 followed the same procedure as Experiment 1, except that the reading digit task was replaced by the spatial fit task. In the spatial fit task, participants made judgements about whether the length of the horizontal bar was short enough to fit the gap between the two squares (Fig 5). In each trial, participants were visually presented with increasingly longer lists of two to eight consonants, and the presentation of the letters was interleaved with four spatial fit judgements. Participants should respond to the spatial fit via mouse clicks (right button for “Yes, it fits” and left button for “No, it does not fit”). We glued coloured stickers (green for “Yes” and red for “No”) on the mouse buttons to remind participants of their meaning. The word “Rappel” (“recall” in French) was displayed on the screen at the end of the trial to prompt participants to orally recall the list of letters. After responding, the participant themself controlled the release of the next trial via mouse click. As in Experiment 1, the length of the lists increased by one letter at every three trials. The task was interrupted when all three lists at any given length were erroneously recalled.

We manipulated the CL of the task by varying the pace of presentation of the spatial fit. In the control condition, each spatial fit was displayed for 1500 ms with 500 ms of interstimulus interval. In the individualized condition, the duration of the spatial fit task was adapted according to each participant’s mean RT, previously calculated at the beginning of the experimental session. In the adapted condition, each stimulus for the spatial fit task was displayed for 1.5x(mean RT) ms. Responses via mouse clicks were enabled only during the display of the spatial fit stimulus and the mouse clicks did not prompt the onset of the next stimulus. After four fit space judgements, the next letter in the list was displayed.

The calculation of the mean RT was done at the beginning of the experimental session. Participants were told that the experiment started with a “challenge” in which they should score 10 points in the spatial fit task in order to proceed to a second phase including the memorization of letters. A score was displayed on the upper right corner of the screen during this stage. Participants were given one point each time they gave a correct response to a spatial judgement, i.e., whenever they responded “Yes” when the gap between the two squares was large enough to accommodate the horizontal bar, and “No” in the opposite case. This phase of the procedure ended when participants scored 10 points. In this stage, the spatial fit stimulus was displayed for 1000 ms and was followed by a blank screen until a response was made. Mouse responses prompted the onset of the next spatial fit stimulus. There was a 500 ms interval of a blank screen between the participant’s response and the onset of the next stimulus. In case participants responded faster than 1000 ms, the mouse clicks prompted the offset of the stimulus. The mean RT in correct trials was later used as a temporal parameter in the individualized pace condition.

Following the training of the spatial fit task and the RT assessment, the experimenter informed the participant that the task would become more difficult because they had a time limit to respond to the spatial fit task. In this new stage, the participant scored zero if they did not respond on time. After receiving these instructions, the participant was trained in one practice trial including a sequence of two letters and then the block of experimental trials began.

We manipulated the CL condition between blocks of trials and the order of presentation of blocks was counterbalanced between participants. The experimenter prompted the program to proceed to the next condition block when the stop rule of three consecutive erroneous trials of the same length was reached in the first block of trials. At that point, the participant was told that the pace of the spatial fit task was going to change. The participant then performed one practice trial of two letters in the new pace condition. Participants with ADHD were allowed small breaks during the task, usually 10 minutes between each block condition.

### Data analysis

We analysed the mean span, the percentage of letters correctly recalled, and the CL. The means of these variables were compared across participants and pace conditions using Bayesian repeated-measure ANOVAs with the group as a between-factor and the pace as a within-factor. For the sake of brevity, we only present the results of the mean span and the CL. The analysis of the percentage of correct letters was reported in the supplementary materials.

### Results

#### Mean spans

**Fig 6:**
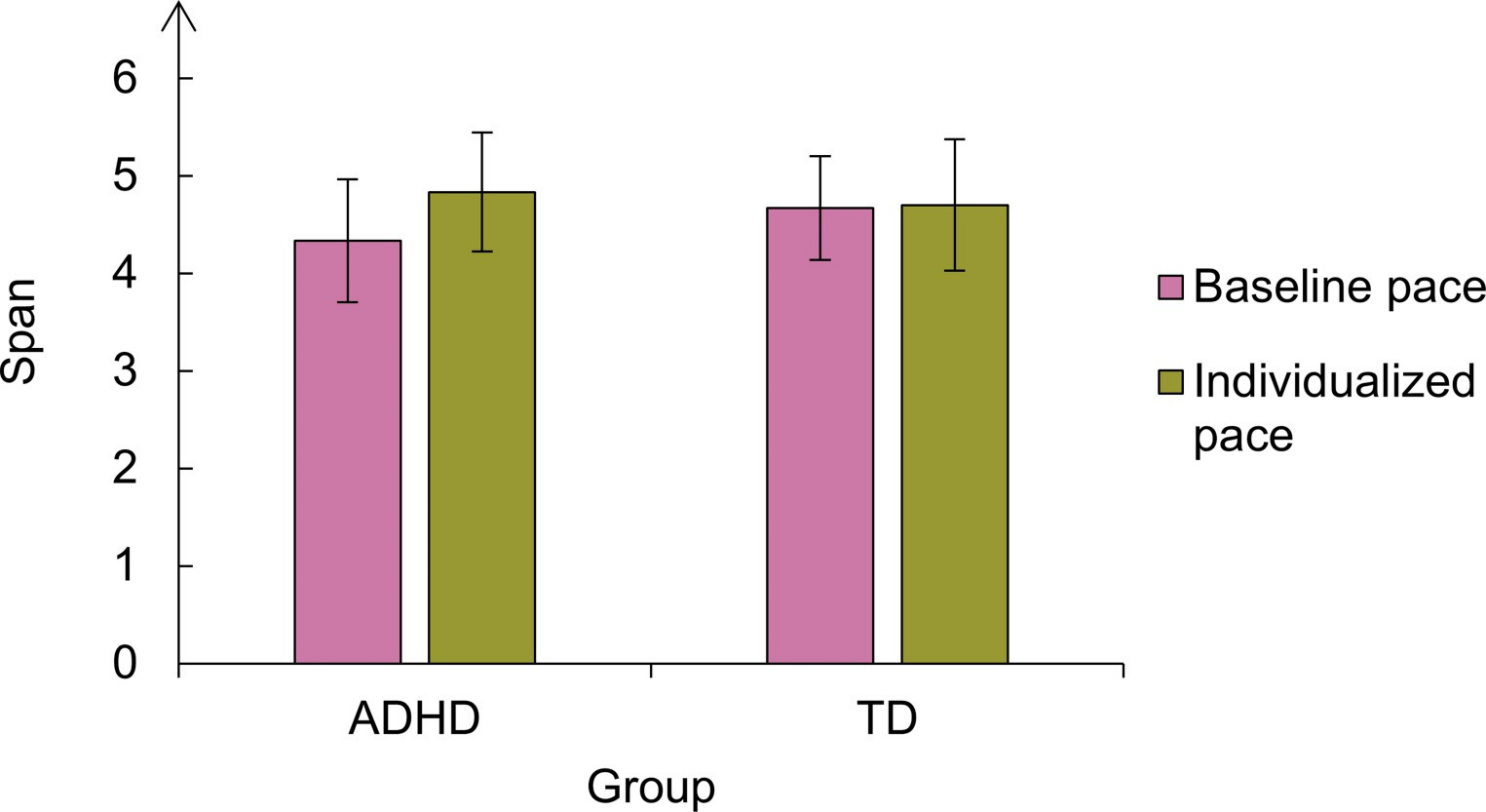
Mean spans per group and condition in Experiment 2. The vertical lines represent the confidence intervals.

In the ADHD group, the mean span was 4.3 (*SD* = 1.1) in the baseline pace and 4.8 (*SD* = 1.06) in the individualized pace. In the control group, the mean span was 4.7 (SD = 0.9) in the baseline pace and 4.7 (*SD* = 1.1) in the individualized pace (Fig 6). According to the Bayesian ANOVA, the best model accounting for the data was the null. We found no evidence in favour of a pace effect (BF_10_ = 0.444, error = 3.51), a group effect (BF_10_ = 0.35, error = 1.42), nor their interaction (BF_10_ = 0.15, error = 1.53). Accordingly, there was no evidence for the inclusion of these factors nor their interaction, with all BF_incl_ between the range of 0 and 0.5.

**Fig 7:**
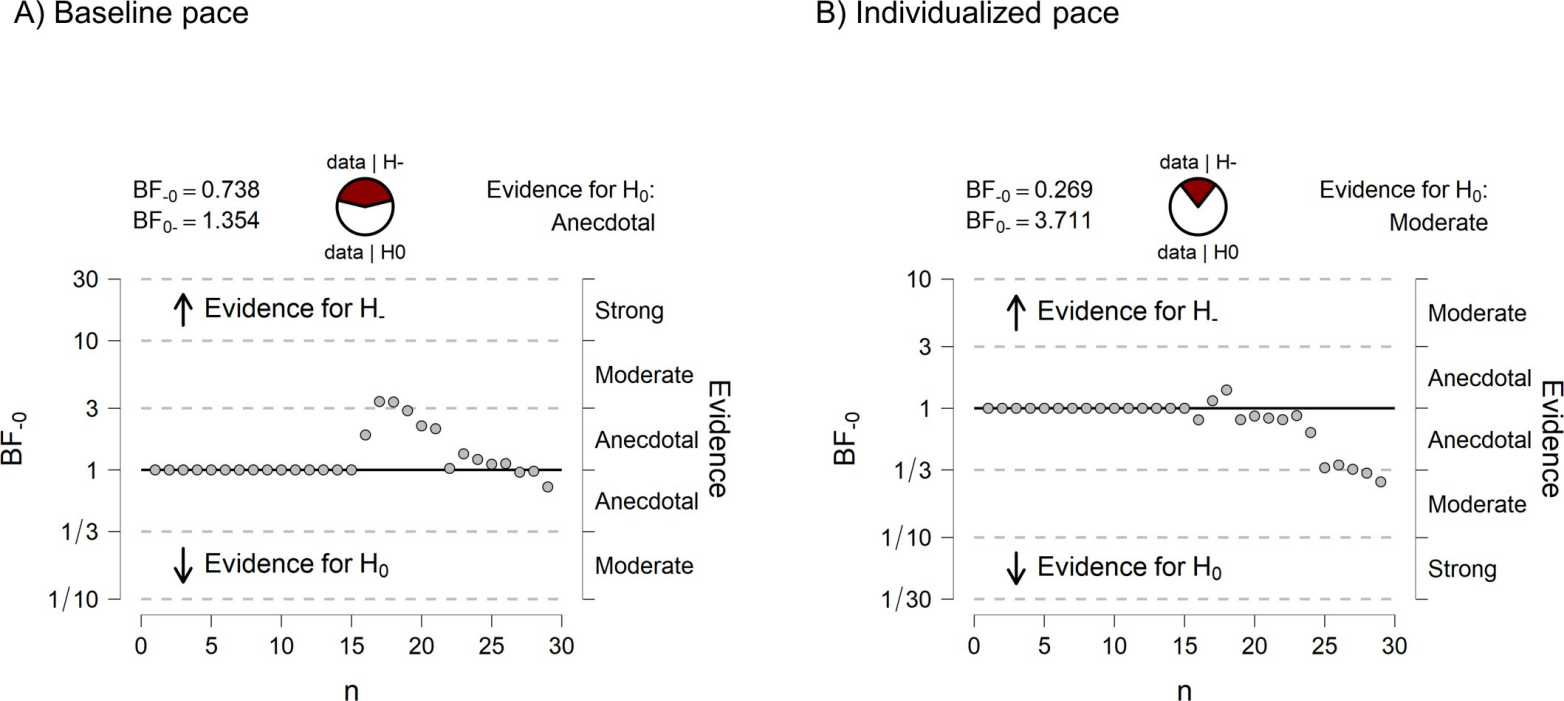
Sequential Bayesian analysis of the mean spans in Experiment 2. The panels reproduce the output from Jasp (Jasp Team, 2022) The x-axes in the plots represent the participant number and the y-axes represents the magnitude of the BF_10_ at every new addition of a data point in the analysis. A BF_10_ = 1 means that the null and the alternative hypothesis are at odds. The horizontally aligned dots until n = 14, on the left side of each plot, correspond to the BF_10_ after the addition of data in the ADHD group. From n = 15 onwards, the dots represent the cumulative changes in the BF_10_ after the addition of data in the control group.

A sequential Bayesian analysis comparing the two groups with *T*-tests showed that there was no trend toward a group difference (Fig 7). These results suggest that implementing the individualized pace in Experiment 2 did not affect memory recall in any of the groups, contrary to our hypothesis.

**Fig 8:**
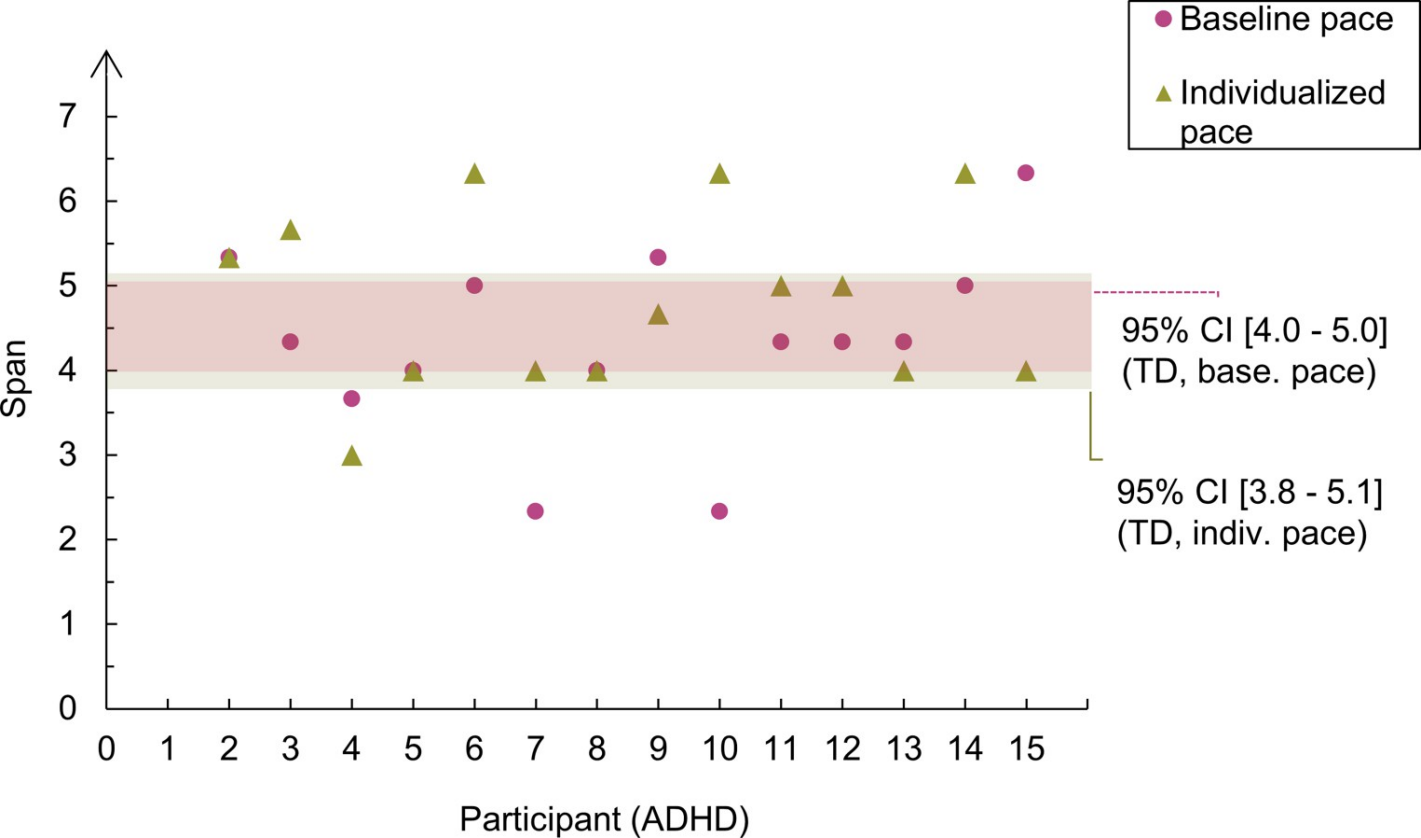
Individual spans in the ADHD group per pace condition in Experiment 2. Each data point in the graph represents an individual span in the ADHD group. The shaded zones represent the 95% confidence intervals of the mean in the control group.

A closer inspection of the individual spans (Fig 8), showed that performance was very heterogeneous in the ADHD group, including in the individualized pace. Some participants greatly benefitted from the individualized pace (e.g., participant 10), while others were not affected (participants 6 and 8) or even disrupted by it (participant 15). This high variability in the mean spans suggests that our pace manipulation affected the CL differently for each participant. Following, we present the analysis of the CL of the task to clarify the effect of our pace manipulation upon the results.

#### Cognitive loads

The absence of a pace effect in Experiment 2 critically contrasts with the results of Experiment 1, in which we found extreme evidence that our pace manipulation increased the CL, leading to a drop in recall memory. For this reason, we ran a post-hoc analysis to examine whether the adaptation of the pace in Experiment 2 effectively changed the CL of the task in the individualized condition. We calculated the individual CLs of participants in the two pace conditions of Experiment 2. In the baseline pace, the CL was calculated by dividing the mean RT of correct responses in the spatial fit (*Ta*, time of attentional capture by the concurrent task) in the spatial fit task by 2000 ms (*Tt*_(normal)_, the total time between each spatial fit, including the 500 ms interstimulus-interval). In the individualized pace, the CL was calculated by dividing the mean RT of correct responses in the spatial fit task (*Ta*) by the sum of the adapted stimuli duration and 500 ms interstimulus interval (*Tt*_(adap)_ = Adapted duration + 500 ms).

**Fig 9:**
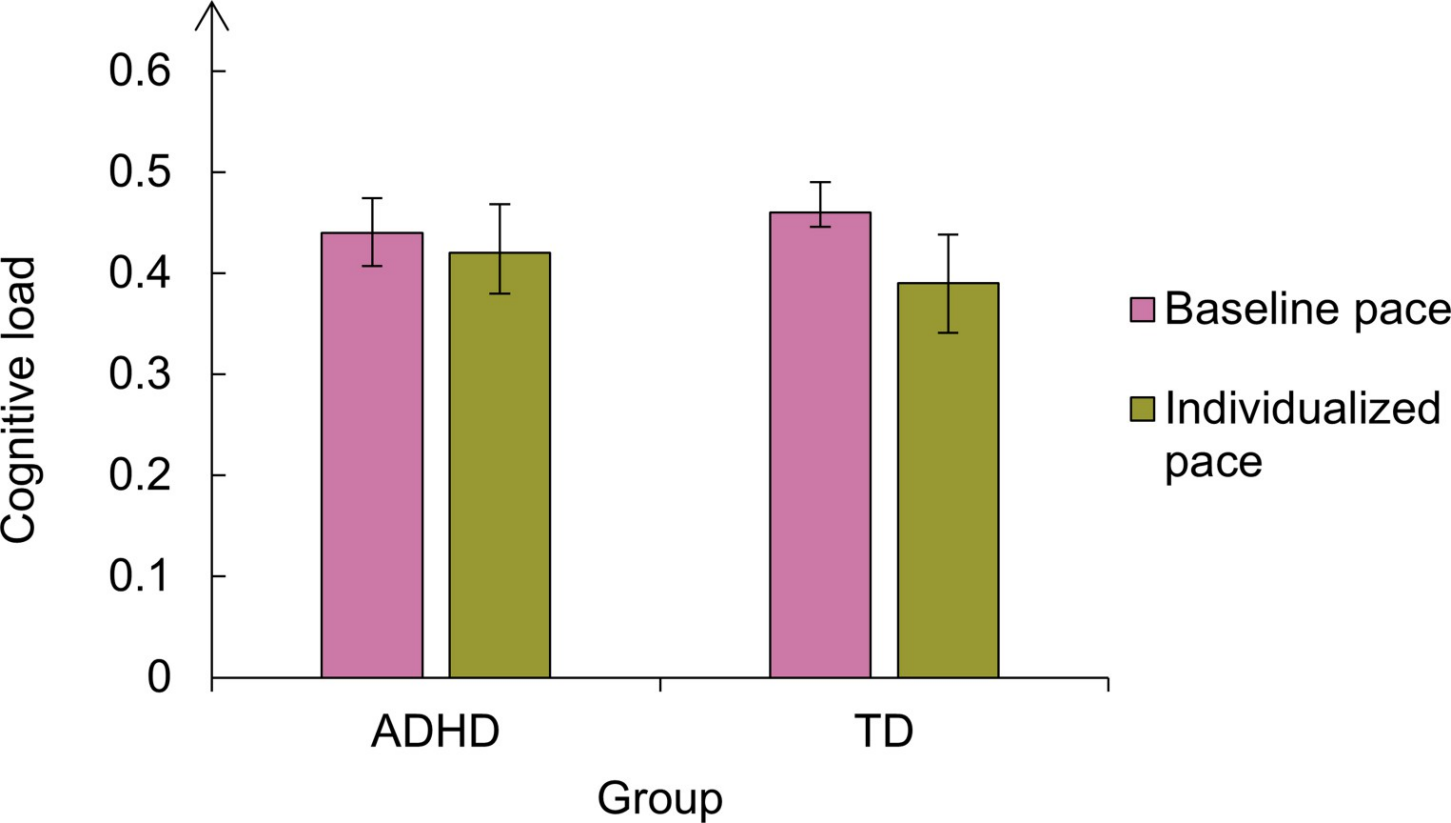
Mean cognitive load per group and condition in Experiment 2. The vertical lines represent the confidence intervals.

The individualized pace in Experiment 2 could have led to small variations in the CL that did not convert to real gains in memory recall. Alternatively, it is also possible that the individualized pace had no impact upon the CL of participants at all. We compared the CLs across groups and pace conditions with a Bayesian repeated-measures ANOVA to rule out these accounts of the results. As expected, the individualized pace led to a reduced CL compared to the baseline CL. The mean CL in the ADHD group was 0.44 (*SD* = 0.06) in the baseline pace and 0.42 (*SD* = 0.07) in the individualized pace. The mean CL in the control group was 0.46 (*SD* = 0.03) in the baseline pace and 0.39 (*SD* = 0.08) in the individualized pace. Fig 9 shows the mean CL in each group and experimental condition. A table containing the individual CLs per participant can be found in the supplementary material.

The best model accounting for the data was the one including only the main effect of the pace (BF_10_ = 19.35, error = 1.07). The second-best was the full model, including the main effects of the pace, the group, and their interaction (BF_10_ = 16.79, error = 2.37). We found strong evidence in favour of the inclusion of the pace (BF_incl_ = 21.73), but not for the inclusion of the group (BF_incl_ = 0.8, no evidence) and its interaction with the pace (BF_incl_ = 2.38, anecdotal evidence). Our interpretation of the Bayesian analysis is that the individualized pace yielded small, but consistent decreases in the CL across our sample, but this change in the CL was not followed by an increase in the spans as predicted by our hypothesis.

#### Comparison between Experiment 1 and Experiment 2

The absence of a beneficial effect of the individualized pace in Experiment 2 contradicts the observation of a strong deleterious effect of the fast pace in Experiment 1. The two experimental designs are complementary manipulations of the pace and follow the same rationale. In Experiment 1, the addition of a fast condition intended to hamper performance by increasing the CL of the task. Complementarily, the individualized pace condition in Experiment 2 aimed at improving recall by decreasing the CL. Moreover, we kept the pace of the presentation of stimuli in the slow condition (Experiment 1) and in the baseline condition (Experiment 2) identical, with a ratio of 0.33 between the free time (blank screen) and the duration of the stimuli of the processing task, so that the two conditions should be comparable between experiments. The diverging results in Experiments 1 and 2 raise the question of why recall performance was hampered by the fast pace but not improved by the individualized pace.

We examined the effects of the pace manipulation in Experiments 1 and 2 by running Bayesian repeated-measures ANOVAs to compare the differences in the mean spans between the two levels of the pace (slow/fast vs. baseline/individualized) in each group. In the ADHD group, we set the factors experiment (Experiment 1 vs. 2) and pace (slow/normal vs. baseline/individualized) as within factors because participants took part in the two experiments. In the control group, we set the experiment (Experiment 1 vs. 2) as a between-factor and the pace (slow/normal vs. baseline/adapted) as a within factor because each participant took part in only one experiment.

**Fig 10:**
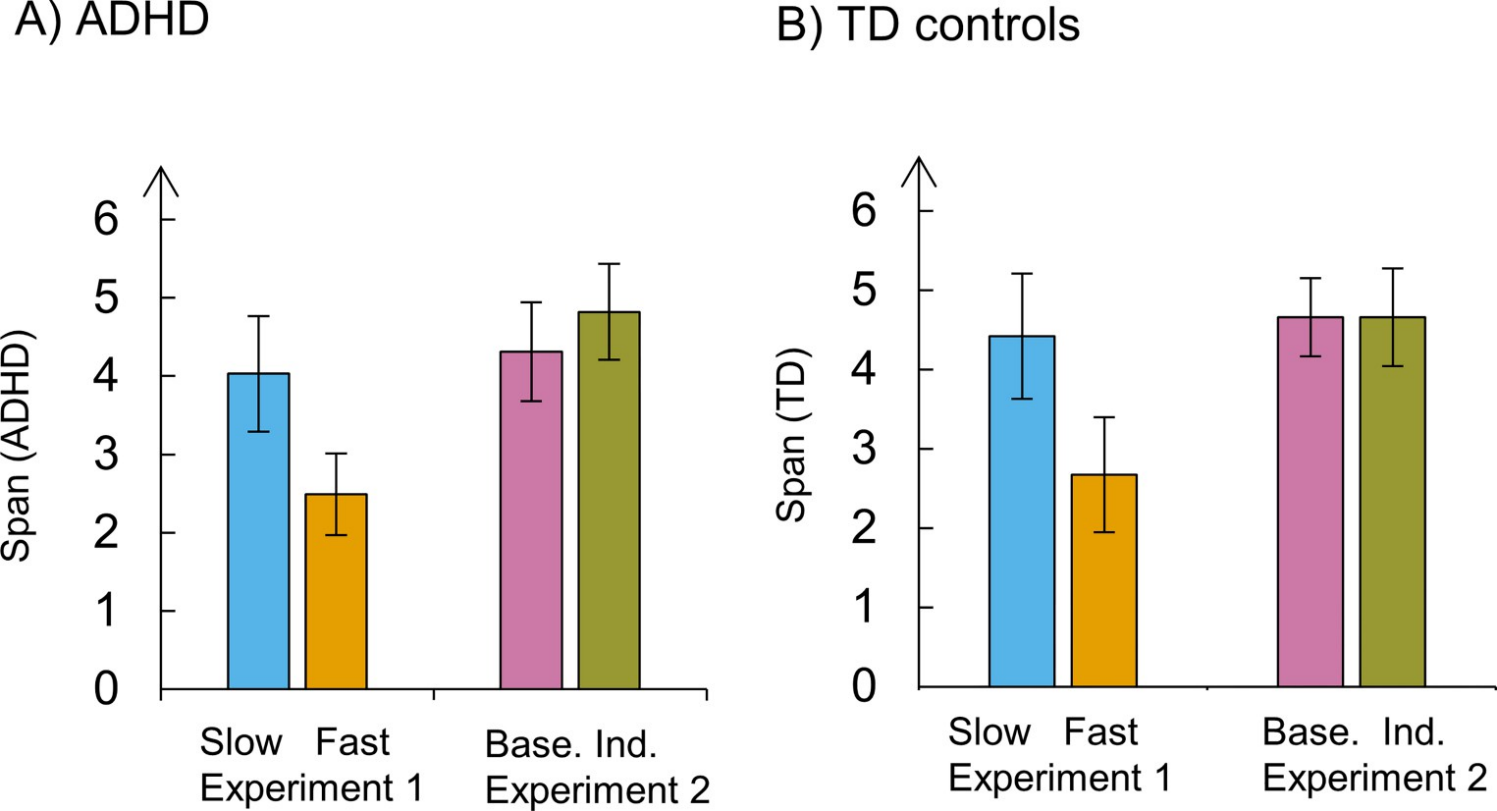
Comparison between the effect of the pace manipulation in Experiments 1and 2 for each group of participants. The vertical lines represent the confidence intervals.

Both for ADHD and controls, the best model accounting for the data includes the main effects of the experiment, the pace, and their interaction. In the ADHD group, the Bayes factor of the full model was BF_10_ = 7.09 × 10^4^ (error = 2.26) and we found decisive evidence for the inclusion of the factors experiment (BF_incl_ = 2.677 × 10^4^), pace (BF_incl_ = 977.3), and their interaction (BF_incl_ = 3.119 x 10^3^) in the model. In the control group, the Bayes factor of the full model was BF_10_ = 1.649× 10^4^ (error = 1.73) and we found decisive evidence for the inclusion of the factors experiment (BF_incl_ = 229.25), pace (BF_incl_ = 2.201 × 10^3^), and their interaction (BF_incl_ = 240.84) in the model. It is noteworthy that, among ADHD participants, the strongest evidence was for the inclusion of the experiment (BF_incl_ = 2.677 × 10^4^), whereas, among controls, the strongest evidence was in favour of the pace (BF_incl_ = 2.201 × 10^3^). This difference in the BF_incl_ between ADHD and controls suggests that the pace effect in ADHD participants was more variable depending on the experiment. Fig 10 illustrates the effects of our pace manipulations upon the mean spans across experiments and groups of participants.

In Experiment 1, the fast pace clearly impaired recall performance of both ADHD (two leftmost bars in the plot, Fig 10A) and controls (idem, Fig 9B), causing a decrease in the mean span. However, in Experiment 2, ADHD participants showed a trend for improvement in performance in the individualized pace (two bars to the right, Fig 10A), whereas controls were unaffected by it (idem, Fig 10B). The comparison between Experiment 1 and Experiment 2 confirmed that the effects of the manipulation of the pace were different between the two tasks, and that this difference was modulated by the factor group. These results contributed to the inclusion of the factor group and the interaction group x experiment in the model. In sum, the effect of our pace manipulation was different across experiments and more variable in the ADHD group. Following, we will present RT data that help to explain why we found divergent effects of the CL between Experiments 1 and Experiment 2.

#### Response times

As explained in the introduction of Experiment 2, the basis of the CL effect lies in differences in processing speed that are indexed by the RTs. When participants are faster to process a secondary task during a limited interval, their attention is free to refresh the memorized items until the end of the interval, i.e., until the next letter is presented. To assess how the pace manipulation affected participants’ RT and better understand its effects on the CL, we ran a 3x2 Bayesian repeated-measures ANOVA. We set the condition (training, baseline, individualized) as a within-participant factor and the group (ADHD, control) as a between-participant factor. We included the RTs during the training in this analysis because we used this parameter to set the pace in the individualized condition. Hence, we wanted to know whether the mean RT assessed in the training phase was kept constant by participants during the other two blocks of trials.

In the ADHD group, the mean RT was 1352 ms (*SD* = 50) in the training, 881 ms (*SD* = 11) in the baseline, and 1001 ms (*SD* = 233) in the individualized condition. In the control group, the mean RT was 1548 ms (*SD* = 899) in the training, 936 (*SD* = 79) in the baseline, and 997 ms (*SD* = 162) in the individualized condition. Overall, participants were slower in the training than in the baseline and the individualized condition. The best model accounting for the data was the one including only the main effect of the condition (BF_10_ = 1.0 x 10^4^), and this is explained by participants being slower in the training phase. Regarding potential group differences, although ADHD participants tended to be faster than controls during the training and the baseline condition, we found no evidence for the inclusion of the factor group in the model (BF_incl_ = 0.286).

The analysis of the RTs suggests one main reason why we did not find the expected CL effect in Experiment 2. Slower RTs in the assessment phase indicate a training effect that caused participants to be faster in the baseline and individualized blocks. In the baseline condition, the duration of each spatial fit judgement was 1500 ms, which was much superior to the mean RT of both groups in this block of trials. In the individualized condition, the duration of the spatial fit judgement exceeded participants’ RTs even more, since it was defined as 1.5 x RT_(training)_ and participants were slower during the training. The time available to perform the spatial judgement task both in the baseline and individualized conditions was, therefore, more than enough for participants to do the spatial judgement task without the adaptation of the pace to improve their span. In other words, our participants were not constrained enough in the baseline condition in order to benefit from the individualization of the pace. This ensemble of results reinforces our interpretation that there was no room for improvement in the individualized condition and explains why the alleviation of the CL did not improve performance in Experiment 2.

### Discussion

The fundamental assumption behind the design of Experiment 2 is that adapting the pace of presentation of the processing component of the task (i.e., the spatial fit task) to each participant’s mean RT would equalize the CL across participants and control for individual differences in processing speed. Our reasoning follows the predictions of the TBRS model of WM, according to which any manipulation of the pace of the processing component of a complex span task will cause changes in the cognitive load (CL), thus affecting recall performance. Indeed, we observed this prediction in Experiment 1, in which the fast pace of the reading of digits led to an increase in the CL and a consequent drop in performance. Nevertheless, the results of Experiment 2 showed that the adaptation of the pace of the spatial fit task caused no changes in recall performance in both groups of participants, contrary to our hypothesis.

An important question is why the manipulation of the pace did not affect recall performance in Experiment 2, whereas it had a strong effect in Experiment 1. A straightforward explanation is that the individualized pace had a very heterogeneous effect in our ADHD group, with some participants greatly benefitting from it and others being unaffected or even disrupted by it (Fig 8). Another explanation is that the task in Experiment 2 did not obliterate the use of phonological rehearsal like the reading digit task in Experiment 1. This would mean that participants could use this maintenance strategy and be less reliant in attentional refreshing to maintain letters in Experiment 2. Finally (and not exclusive to the other two explanations presented), participants were performing near ceiling in the baseline condition in Experiment 2, with mean spans very close to the ones observed in the slow condition in Experiment 1. This suggests that the time parameters we used in the baseline pace were not constraining enough to make participants need the individualized pace to achieve their best recall performance. If participants were already performing at their best (which implies optimum use of attentional refreshing), having more time available to refresh the items will not cause any improvement in recall.

Despite the absence of a pace effect upon the mean span, we found strong evidence that the individualized pace caused a slight decrement in the CL in Experiment 2, according to our prediction. In other words, adapting the pace of presentation of the stimuli in the processing task alleviated the CL for participants, but it did not promote an improvement in memory recall. The results of our Experiment 2 call for replication and encourage further investigations, possibly with some modifications in the implementation of the tasks, e.g., by modifying the time parameters of the baseline pace to avoid a ceiling effect.

## General discussion

This study was the first in the literature to examine the CL effect and the use of attention to maintain information in verbal WM in children with ADHD symptoms. To this aim, we implemented two complex span tasks manipulating the pace of the processing component to vary the CL and tackle attentional refreshing, a pivotal maintenance strategy described by the TBRS model of WM. As explained in the introduction, the CL effect is an index of the use of attentional refreshing during complex span tasks. We had two main predictions. First, the CL effect should be observed in our sample in case children and adolescents with ADHD symptoms use attention for maintenance purposes. Second, the slope relating the mean spans to the CL should be less steep in the ADHD group than the one reported in the literature for typically developing individuals [21], in case attentional refreshing is less efficient in ADHD participants. We observed a strong effect of our pace manipulation on the spans in Experiment 1 and on the CLs in Experiment 2, both in ADHD and control participants. In Experiment 1, the fast pace caused an increase in the CL of the task and a consequent decrease in the mean spans. In Experiment 2, the adaptation of the pace according to each participant’s RT caused an alleviation in the CL, but it did not affect the mean spans.

In Experiment 1, both ADHD and typically developing participants had poorer recall memory in the fast pace than in the slow pace condition. This result suggests that the temporal constraint of reading digits in the fast pace increased the CL, thus preventing participants from using attentional refreshing to maintain the letters. We highlight the fact that we observed a strong CL effect both in ADHD and control groups, with no group differences. Even with a relatively small and heterogeneous sample of children with ADHD symptoms, the curves relating the CL to memory performance behaved as predicted by the TBRS model and similarly to previous reports in the literature. Moreover, our results are aligned with a previous result in a visuospatial complex span task [37], in which recall memory in children with ADHD was also prone to a manipulation affecting the CL of the task. Taken together, these results suggest that attentional refreshing can be used both during visuospatial and verbal tasks, as observed in typically developing children. We stress that our participants were not instructed to use attentional refreshing, therefore the observation of the CL effect in Experiment 1 shows the spontaneous use of this strategy by children with ADHD. Despite their attentional deficit, children diagnosed with ADHD seem to use attention to maintain information in WM in the short term. Therefore, their WM functioning is prone to the same time constraints observed in typically developing individuals.

WM maintenance happens via two main mechanisms. Besides attentional refreshing, which is an attention-demanding and domain-general strategy, articulatory rehearsal is not attention-demanding and is specifically dedicated to maintaining verbal information [42, 43]. Intuitively, one may consider that children suffering from an attention deficit would not be able to use attentional refreshing for maintaining information and would systematically favour the use of articulatory rehearsal, which is not attention-demanding. One may even suggest that they would neglect to maintain information in WM and not employ any of the two strategies, relying solely on some episodic traces stored in long-term memory (LTM) to recall. The latter possibility is contradicted by the emergence of a CL effect on recall performance in the present study. For a comparison, young typically developing children (aged 5) wait rather passively until the end of the trial to answer and do not implement any maintenance strategies, basing their responses on LTM traces [21, 32]. For these young children, recall performance is not dependent on the CL of the concurrent task, but only on the delay between encoding and recall [32]. The existence of a CL effect in our sample, therefore, shows that our participants are actively engaged in a maintenance strategy requiring attentional resources.

Regarding Experiment 2, as explained, our manipulation of the pace caused a slight, but significant, drop in the CL that was not followed by an increase in recall performance, both in ADHD and control participants. This result is inconclusive and contradicts the observation of a strong pace effect upon recall performance in Experiment 1. We suggested previously that our participants probably relied more heavily on articulatory rehearsal in Experiment 2 than in Experiment 1, in which the concurrent processing task involved verbal information and thus depleted the use of articulatory rehearsal. The parallel use of articulatory rehearsal and attentional refreshing in Experiment 2, together with the ceiling effect in the baseline condition, can account for the differences between the two experiments. This interpretation is in alignment with the proposal of the TBRS model that verbal information is maintained in WM via both attentional refreshing and articulatory rehearsal [43, 44].

Our results suggest that the deficits in WM performance observed in children with ADHD are not caused by an inability to use attentional refreshing. Alternatively, a developmental delay in the acquisition and optimization of this strategy could be another potential source of WM deficits in ADHD. The use of attentional refreshing emerges in typically developing children around age 7 and is optimized until adolescence [21, 22, 32]. Our study did not test different age groups to assess the development of this WM mechanism in ADHD, but it can indirectly inform us about a developmental trend. The absence of group differences in our results suggests that our ADHD participants were not developmentally delayed compared to typically developing controls in the acquisition and use of attentional refreshing. Moreover, the inclusion of the factor age as a covariate in the model suggests that they follow the same developmental trend observed in typically developing children. We encourage future studies to compare different age groups of ADHD children to clarify the hypothesis of a developmental delay.

## Current limitations

A limitation of this study is the size of our sample, which may not be large enough to detect group differences and the group x pace interaction. According to post-hoc power analyses (using the app https://shiny.ieis.tue.nl/anova_power/), in Experiment 1, the power to detect a pace effect was 100%. However, it was only 5.45% to detect a group effect and 26.85% to detect an interaction. In Experiment 2, the power to detect a pace effect was 23.4%, to detect a group effect was 6.9%, and to detect an interaction was 22.4%. The drastic drop in the power to detect a pace effect in Experiment 2 (compared to 100% in Experiment 1) is due to the absence of CL difference between the baseline and individualized conditions, the baseline allowing already a very good recall performance without the need of adapting the CL to the individual processing speed. Given the limited number of participants in our study, further studies should aim at replicating our results with larger samples.

Although our sample was underpowered to detect the hypothesized interaction between pace and group, such interaction was also not observed by Weigard and Huang-Pollock [37] (see Fig 4 of their article), who tested 71 children with ADHD and 27 typically developing controls. They manipulated the difficulty of a numerosity processing task in order to induce a high and low CL and observed a significant effect of this manipulation in both groups. More importantly, they similarly reported an absence of interaction between CL and group. Together, their results and ours suggest that the WM performance of children with ADHD is subject to the same temporal constraints as in typically developing individuals. One of our reviewers pointed out that, with an insufficient sample size, the only way to detect the hypothesized interaction would be in the case that ADHD children did not present the CL effect at all. Such an outcome (i.e., ADHD not being prone to the CL effect) seems unlikely to us in the light of Weigard and Huang-Pollock’s study [37] and based on the fact that the CL effect has been observed in many different age groups and in children as young as 7 years [18, 21–24]. The CL effect, therefore, could reflect a general mechanism of WM functioning, as suggested by Camos et al. [20].

A second limitation is the large variability in the age and symptoms of our participants in the ADHD group. We chose an age group ranging from 10 to 16 years because of evidence that attentional refreshing is a mechanism that reaches adult levels of efficiency during adolescence, around age 14, in typically developing children [21]. There was also a component of necessity in choosing such a large age range, due to difficulties in recruiting participants during the COVID-19 pandemic. The same applies to the inclusion of participants diagnosed with all subtypes of ADHD in our sample. The presence of older participants and participants with lower inattentive symptoms might have masked group differences in our study. Future research should narrow down their age group and, ideally, prioritise the inclusion of participants diagnosed only with the inattentive subtype of ADHD in their sample.

In terms of our method, it is also possible that using a verbal complex span task contributed to the absence of a group effect in our study, although it was a purposeful choice. As we mentioned, Weigard and Huang-Pollock [37] used a visuospatial complex span task with a similar manipulation to ours (i.e., varying the processing speed of participants to affect the CL) and they found group differences between ADHD and controls. Visuospatial WM tasks are more sensitive to the presence of an ADHD diagnosis than verbal tasks, as supported by meta-analytical evidence [9–11]. Our results alone do not surmount all the evidence in the literature showing an ADHD-related deficit in WM. Rather, our sample is small, and it could be that our tasks were not sensitive enough to detect group differences in using attentional refreshing. This is because Experiments 1 and 2 required only memory for verbal content, which can be maintained in parallel via articulatory rehearsal and thus potentially mask a refreshing deficit in ADHD.

Finally, as pointed out by another reviewer, our sample of ADHD participants excluded comorbid neurodevelopmental conditions and therefore might not necessarily reflect the complexity of symptoms and associated traits displayed by this clinical population out in the “real world”. For instance, comorbidities like learning disorders and executive functioning problems are frequent in the ADHD population [45–47], and those conditions might also affect their WM performance and even mask ADHD symptoms [48]. We selected participants presenting exclusively ADHD symptoms because the goal of this research was to find a mechanistic explanation for poor WM maintenance (i.e., misuse of attentional refreshing) in ADHD rather than providing a profile of how these children perform WM tasks in real-life contexts. Including children with comorbid conditions in our sample would be counterproductive to the aims of our experimental manipulation. For instance, comorbidity such as dyslexia could account for poor performance in our experiments because they require memory for letter sequences. Comorbid dyscalculia and its associated difficulties in symbolic number processing and visuospatial WM [49, 50] could impair participants in the ADHD group, particularly less able to perform the reading of the digits (Experiment 1) and the spatial fit (Experiment 2). Moreover, poor WM performance could be caused by comorbid executive problems rather than by ADHD itself. In all the examples given, any group differences between ADHD and controls would not necessarily reflect the purported difficulties in using attentional refreshing.

We advocate caution in interpreting our results as evidence against a WM deficit in ADHD. Rather, they point out that these deficits are not attributable to a misuse of attentional refreshing to maintain verbal information. We encourage future studies to investigate other potential sources of WM deficits in ADHD that are beyond our experimental design, such as a deficient encoding of information into WM [51] and a defective formation of bound representations in WM [52].

## Conclusion

To conclude, this study has shown that children with ADHD are able to use attention for maintaining verbal information in WM. Our experimental design taps the dynamics of attentional refreshing during the maintenance period of a WM task. Thus, our complex span tasks are promising experimental methods to pinpoint WM deficits in individuals with ADHD. More research is required to determine the efficiency of their use of attentional refreshing and how this mechanism develops in children with ADHD compared to typically developing individuals.

## Supporting information

S1 Fig Percentage of letters correctly recalled in Experiment 1 per group and condition. The vertical bars represent the confidence intervals.
(TIF)

S2 Fig Sequential Bayesian analysis of group differences in the percentage of letters correctly recalled in the slow (A) and fast (B) pace of Experiment 1. The panels reproduce the output from Jasp (Jasp Team, 2022). The statistical test used was a *T*-test for independent samples. The alternative hypothesis predicted lower percentages in the ADHD group. The x-axis of each plot represents the participant number and the y-axis represents the magnitude of the BF. Each point in the plot represents a change in the BF caused by the addition of a participant in the analysis. The horizontally aligned dots until n = 15, on the left side of each plot, correspond to data in the ADHD group.
(TIF)

S3 Fig Individual percentages of letters recalled in the ADHD group per pace condition in Experiment 1. Each data point in the graph represents an individual percentage of letters correctly recalled by a participant in the ADHD group. The shaded zones represent the 95% confidence intervals of the mean in the control group.
(TIF)

S4 Fig Percentage of letters correctly recalled by condition and group in Experiment 2. The vertical bars represent the confidence intervals.
(TIF)

S5 Fig Sequential Bayesian analysis of group differences in the percentage of letters correctly recalled in the baseline (A) and individualized (B) pace of Experiment 2. The panels reproduce the output from Jasp (Jasp Team, 2022). The statistical test used was a T-test for independent samples. The alternative hypothesis predicted lower percentages in the ADHD group. The x-axis of each plot represents the participant number and the y-axis represents the magnitude of the BF. Each point in the plot represents a change in the BF caused by the addition of a participant in the analysis.
(TIF)

S6 Fig Individual percentage of letters correctly recalled in the ADHD group plotted against the 95% confidence intervals of the means in the control group in Experiment 2. The zones in light blue and pink represent the confidence intervals of the typically developing controls in the baseline pace and in the individualized pace, respectively.
(TIF)

S1 Table Sample characterization in Experiment 1. Only the variables of interest from the Conners-3 Parent are included in the table. Values between parentheses correspond to standard deviations. Lines in bold represent outliers not included in the analyses.
(DOCX)

S2 Table Sample characterization in Experiment 2. Only the variables of interest from the Conners-3 Parent are included in the table. Values between parentheses correspond to standard deviations. Lines in bold represent outliers not included in the analyses.
(DOCX)

S3 Table Individual CLs in Experiment 2. The cognitive load (CL) of each participant was calculated by dividing the mean RT of correct responses in the spatial fit by the time available to respond to it in each pace condition. Negative values in the column “Difference CL” mean that, for the specified participant, the CL was lower in the individualized pace than in the baseline pace.
(DOCX)
