# Supplementary figures and images for: Can children and adolescents with ADHD use attention to maintain verbal information in working memory?
Source: PLoS One. 2023 Mar 14;18(3):e0282896. doi: 10.1371/journal.pone.0282896 (PMC10013902; doi:10.1371/journal.pone.0282896)

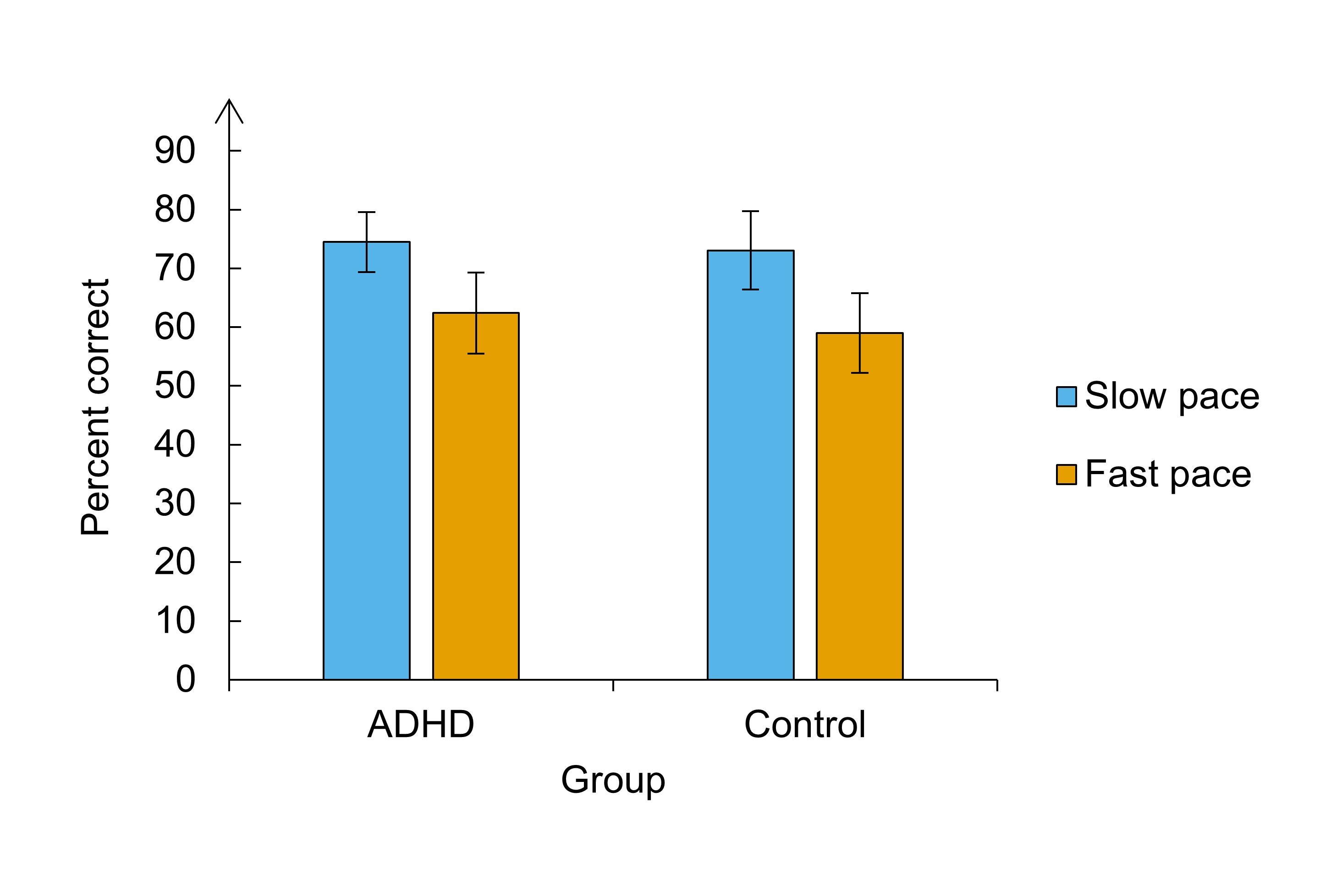

Supplement: S1 Fig — The vertical bars represent the confidence intervals. (TIF) [file pone.0282896.s001.tif]

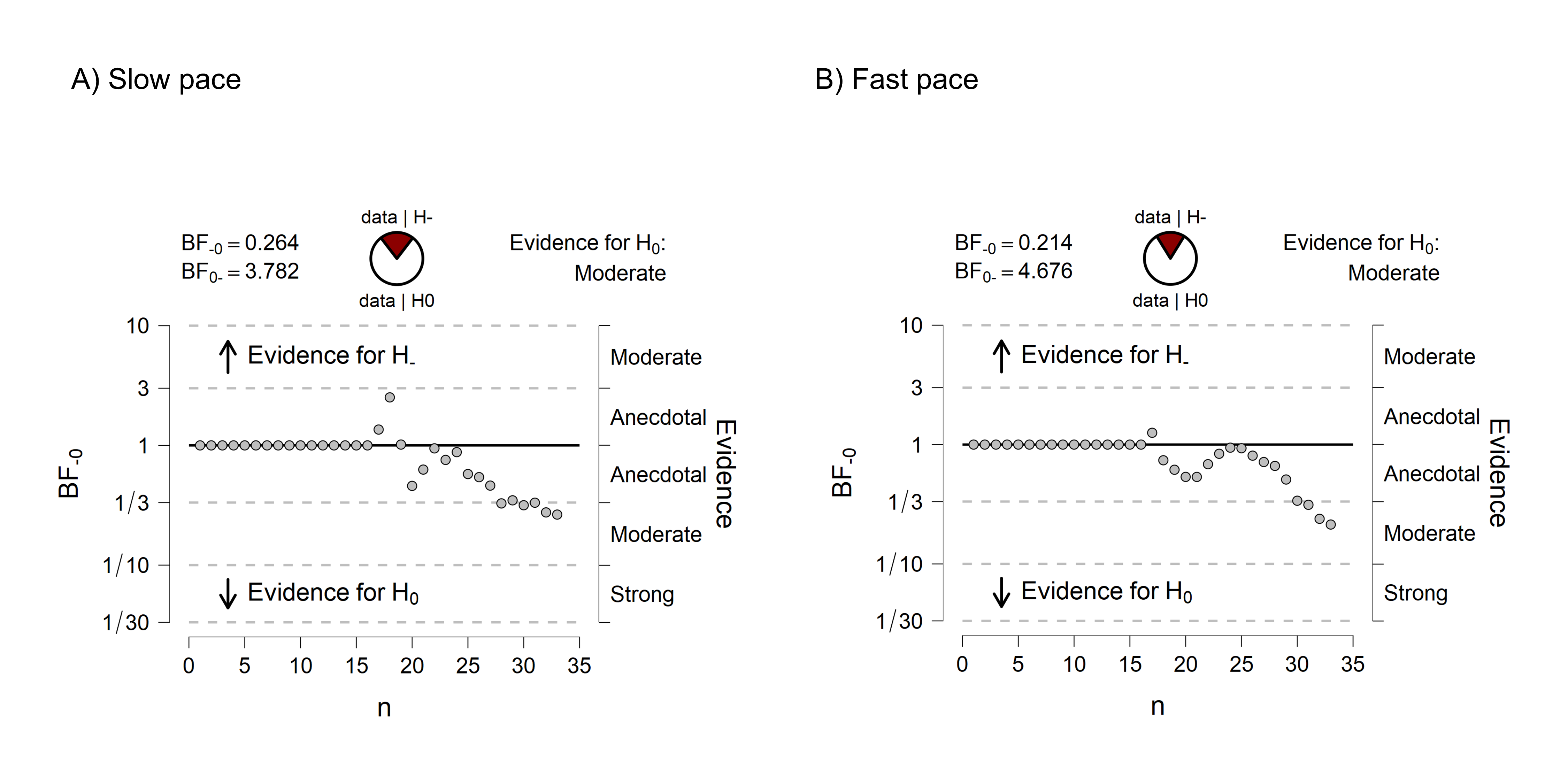

Supplement: S2 Fig — Sequential Bayesian analysis of group differences in the percentage of letters correctly recalled in the slow (A) and fast (B) pace of Experiment 1. The panels reproduce the output from Jasp (Jasp Team, 2022). The statistical test used was a T-test for independent samples. The alternative hypothesis predicted lower percentages in the ADHD group. The x-axis of each plot represents the participant number and the y-axis represents the magnitude of the BF. Each point in the plot represents a change in the BF caused by the addition of a participant in the analysis. The horizontally aligned dots until n = 15, on the left side of each plot, correspond to data in the ADHD group. (TIF) [file pone.0282896.s002.tif]

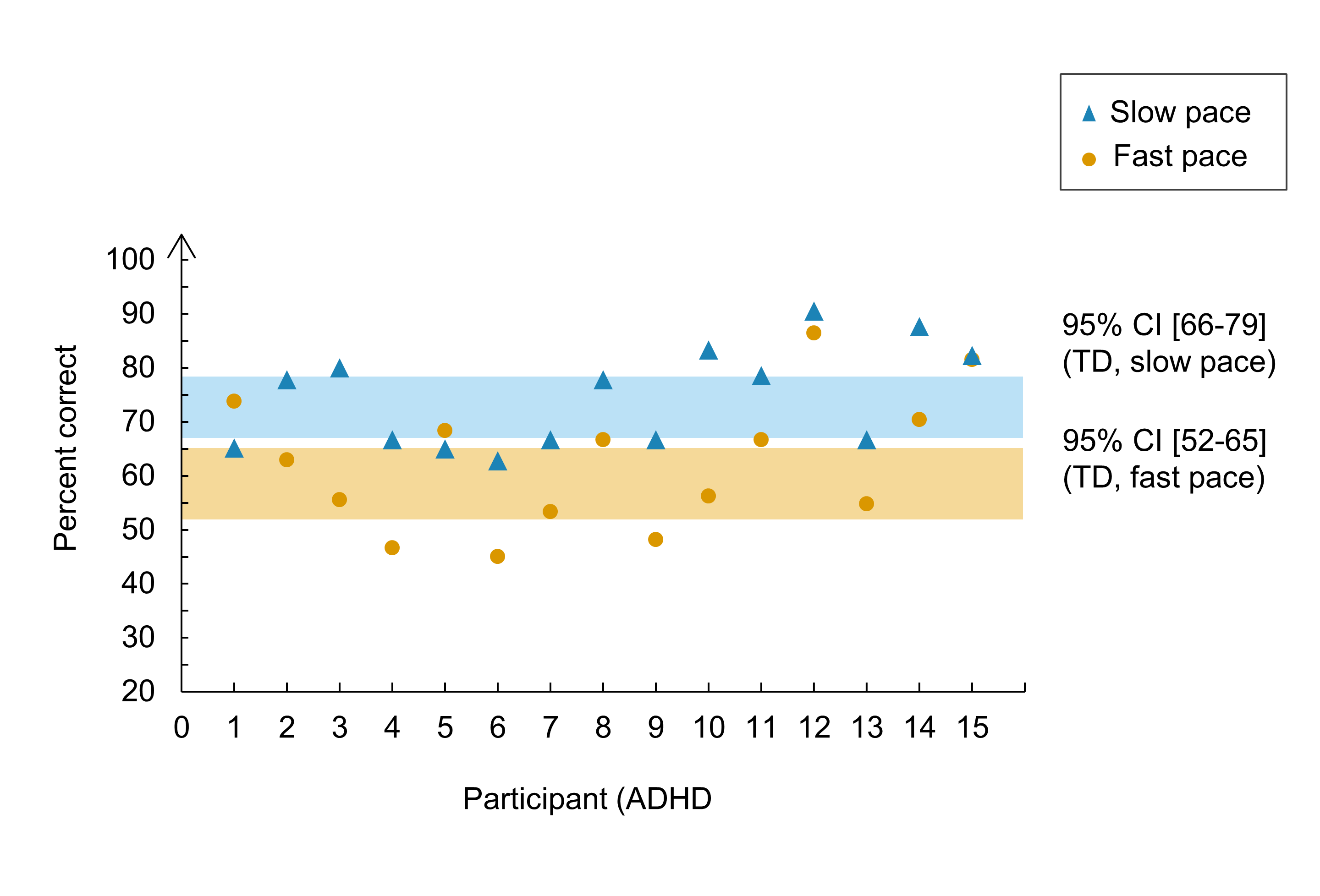

Supplement: S3 Fig — Each data point in the graph represents an individual percentage of letters correctly recalled by a participant in the ADHD group. The shaded zones represent the 95% confidence intervals of the mean in the control group. (TIF) [file pone.0282896.s003.tif]

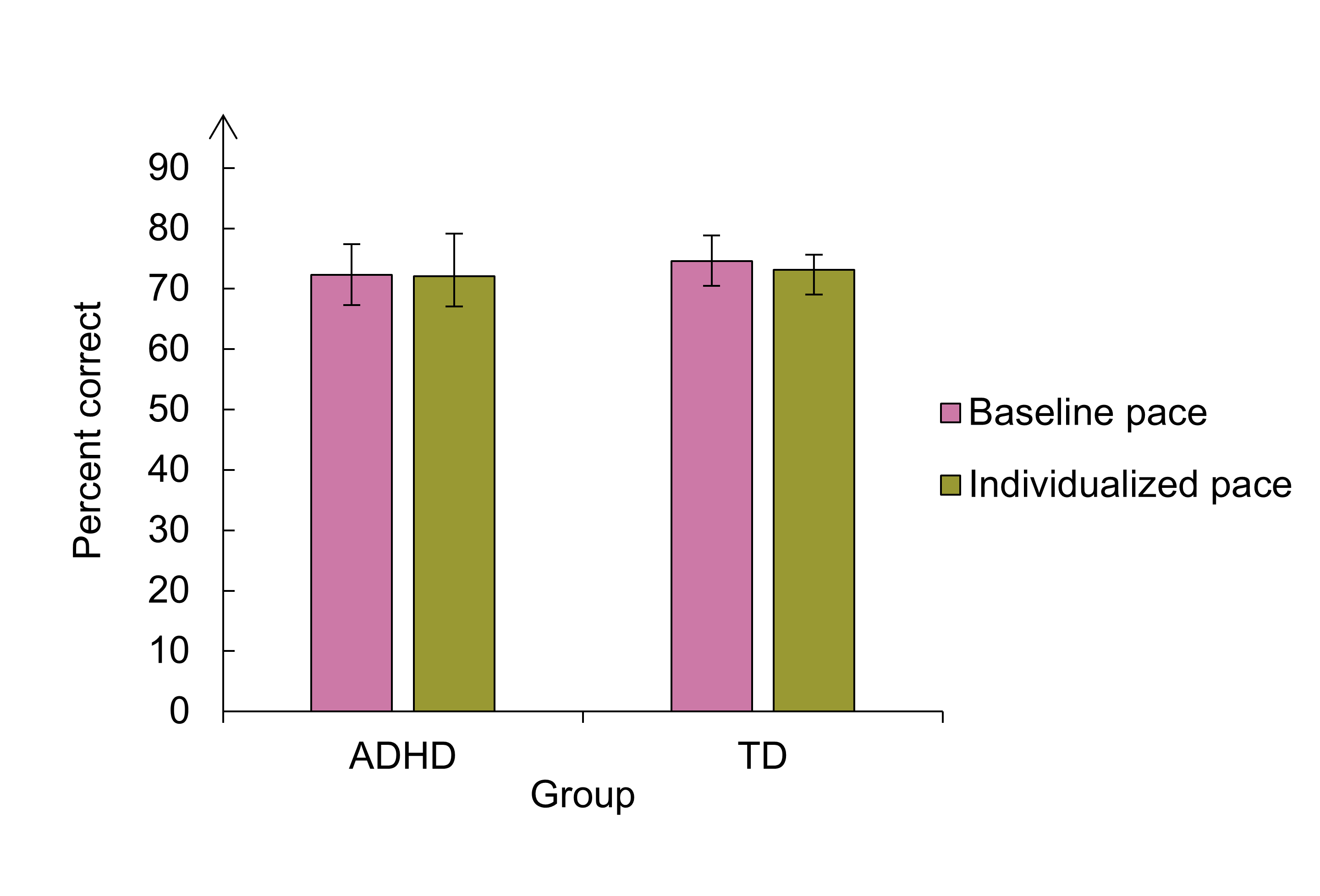

Supplement: S4 Fig — The vertical bars represent the confidence intervals. (TIF) [file pone.0282896.s004.tif]

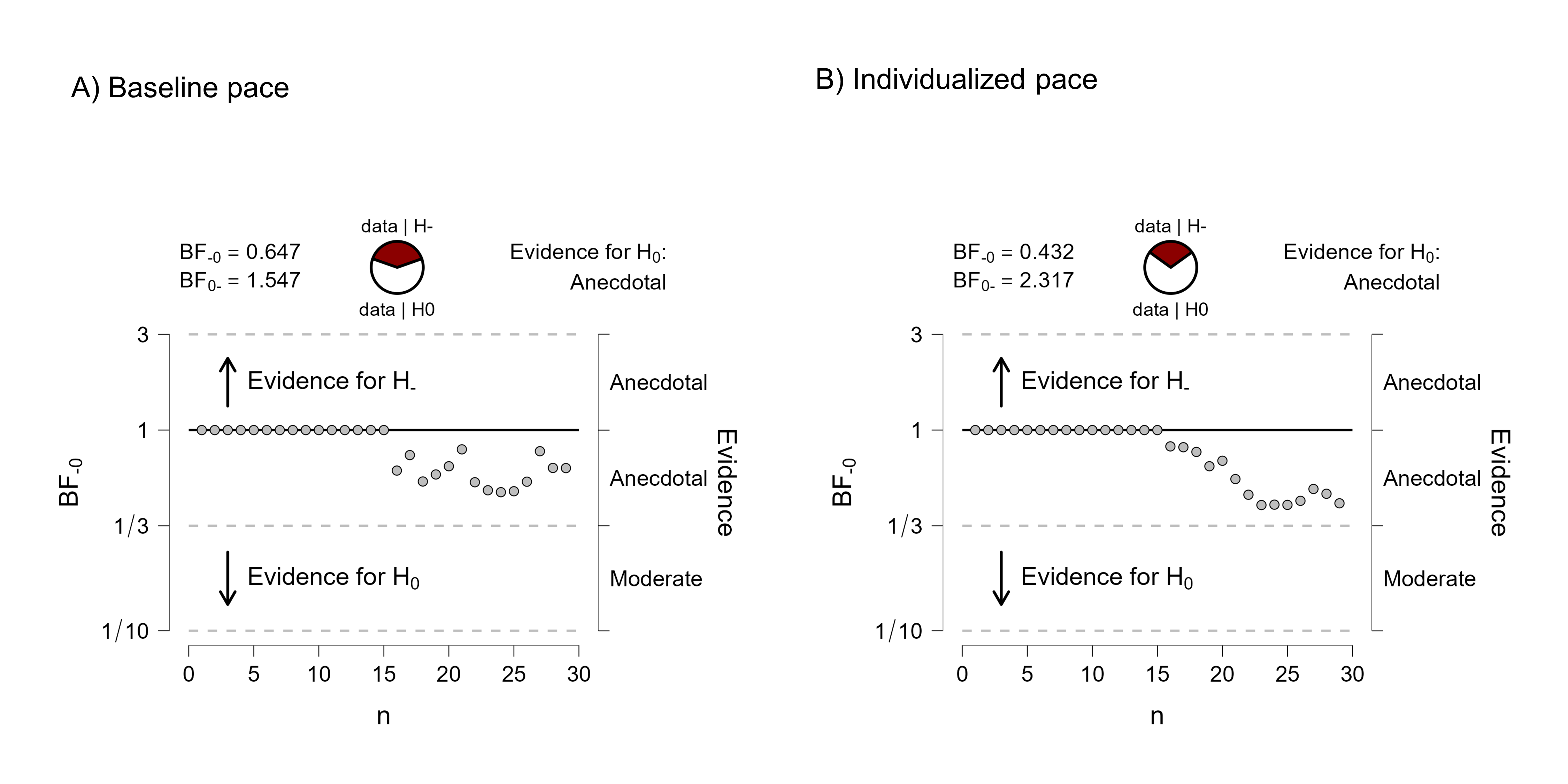

Supplement: S5 Fig — Sequential Bayesian analysis of group differences in the percentage of letters correctly recalled in the baseline (A) and individualized (B) pace of Experiment 2. The panels reproduce the output from Jasp (Jasp Team, 2022). The statistical test used was a T-test for independent samples. The alternative hypothesis predicted lower percentages in the ADHD group. The x-axis of each plot represents the participant number and the y-axis represents the magnitude of the BF. Each point in the plot represents a change in the BF caused by the addition of a participant in the analysis. (TIF) [file pone.0282896.s005.tif]

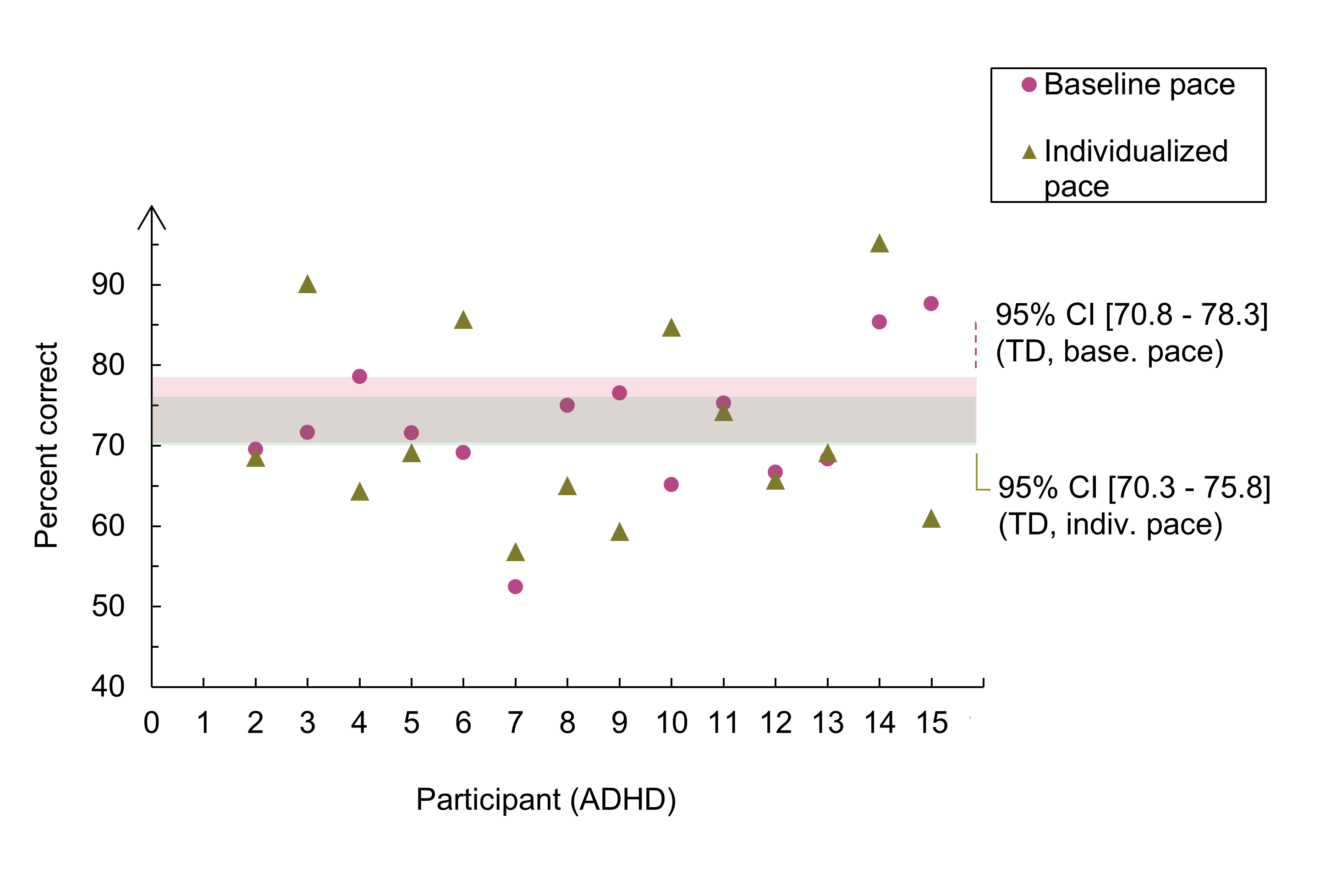

Supplement: S6 Fig — The zones in light blue and pink represent the confidence intervals of the typically developing controls in the baseline pace and in the individualized pace, respectively. (TIF) [file pone.0282896.s006.tif]
