# Supplementary material for: Can children and adolescents with ADHD use attention to maintain verbal information in working memory?
Source: PLoS One. 2023 Mar 14;18(3):e0282896. doi: 10.1371/journal.pone.0282896 (PMC10013902; doi:10.1371/journal.pone.0282896)
Supplement: S1 Table — Only the variables of interest from the Conners-3 Parent are included in the table. Values between parentheses correspond to standard deviations. Lines in bold represent outliers not included in the analyses. (DOCX) [file pone.0282896.s007.docx]

|  | Sex | Age  (years) | ADHD subtype | Inattention | *T*-score  Hyperactive/  Impulsivity | *T*-score  Probability  Index |
| --- | --- | --- | --- | --- | --- | --- |
| ADHD group | F | 11.2 | Combined | 90 | 90 | 0.99 |
|  | F | 15.5 | Hyperactive | 73 | 90 | 0.97 |
|  | F | 12.2 | Inattentive | 90 | 45 | 0.71 |
|  | F | 16.0 | Inattentive | 90 | 47 | 0.87 |
|  | M | 12.9 | Combined | 80 | 72 | 0.99 |
|  | M | 13.3 | Combined | 85 | 89 | 0.99 |
|  | M | 13.5 | Hyperactive | 57 | 67 | 0.41 |
|  | M | 1.7 | Inattentive | 83 | 63 | 0.87 |
|  | M | 15.7 | Combined | 90 | 81 | 0.99 |
|  | M | 11.6 | Combined | 75 | 90 | 0.99 |
|  | M | 13.9 | Combined | 75 | 90 | 0.99 |
|  | M | 13.1 | Inattentive | 66 | 40 | 0.51 |
|  | M | 14.6 | Inattentive | 44 | 45 | 0.11 |
|  | M | 11.9 | Hyperactive | 53 | 67 | 0.56 |
|  | M | 12.7 | Hyperactive | 65 | 90 | 0.91 |
| Subtotals/ Means | 15  (4 females) | 13.2  (1.7) |  | 74.4 (14.7) | 71.96 (19.3) | 0.79 (0.27) |
| Control group | F | 16.0 | - | 52 | 58 | 0.51 |
|  | F | 14.4 | - | 40 | 51 | 0.11 |
|  | F | 12.8 | - | 45 | 42 | 0.11 |
|  | F | 12.7 | - | 40 | 42 | 0.11 |
|  | F | 12.8 | - | 49 | 49 | 0.11 |
|  | F | 10.4 | - | 43 | 53 | 0.11 |
|  | F | 10.9 | - | 45 | 44 | 0.11 |
|  | F | 12.0 | - | 40 | 51 | 0.29 |
|  | F | 11.2 | - | 73 | 64 | 0.64 |
|  | F | 14.6 | - | 64 | 58 | 0.64 |
|  | F | 15.6 | - | 54 | 71 | 0.29 |
|  | F | 10.7 | - | 76 | 44 | 0.71 |
|  | F | 12.6 | - | 68 | 45 | 0.51 |
|  | M | 12.5 | - | 46 | 53 | 0.51 |
|  | M | 15.0 | - | 47 | 41 | 0.11 |
|  | M | 15.4 | - | 40 | 41 | 0.11 |
|  | **M** | **15.7** | **-** | **45** | **68** | **0.41** |
|  | M | 10.9 | - | 59 | 72 | 0.56 |
|  | M | 11.8 | - | 61 | 55 | 0.64 |
| Subtotals/ Means | 19  (13 females) | 13.1 (1.9) |  | 51.9 (11.6) | 52.7 (10.2) | 0.3 (0.23) |
