# Supplementary material for: Can children and adolescents with ADHD use attention to maintain verbal information in working memory?
Source: PLoS One. 2023 Mar 14;18(3):e0282896. doi: 10.1371/journal.pone.0282896 (PMC10013902; doi:10.1371/journal.pone.0282896)
Supplement: S3 Table — The cognitive load (CL) of each participant was calculated by dividing the mean RT of correct responses in the spatial fit by the time available to respond to it in each pace condition. Negative values in the column “Difference CL” mean that, for the specified participant, the CL was lower in the individualized pace than in the baseline pace. (DOCX) [file pone.0282896.s009.docx]

|  | CL (baseline) | CL (individualized) | Difference CL  (indiv. – basel.) |
| --- | --- | --- | --- |
| ADHD group | 0.47 | 0.48 | 0.01 |
|  | 0.41 | 0.42 | 0.01 |
|  | 0.53 | 0.38 | -0.16 |
|  | 0.46 | 0.49 | 0.02 |
|  | 0.35 | 0.26 | -0.09 |
|  | 0.32 | 0.43 | 0.11 |
|  | 0.42 | 0.39 | -0.02 |
|  | 0.42 | 0.37 | -0.05 |
|  | 0.43 | 0.36 | -0.07 |
|  | 0.44 | 0.40 | -0.04 |
|  | 0.50 | 0.47 | -0.02 |
|  | 0.50 | 0.42 | -0.08 |
|  | 0.48 | 0.58 | 0.10 |
|  | 0.44 | 0.48 | 0.04 |
| Control group | 0.40 | 0.39 | 0.00 |
|  | 0.45 | 0.43 | -0.02 |
|  | 0.48 | 0.38 | -0.10 |
|  | 0.49 | 0.43 | -0.06 |
|  | 0.44 | 0.20 | -0.24 |
|  | 0.48 | 0.37 | -0.11 |
|  | 0.45 | 0.43 | -0.02 |
|  | 0.50 | 0.37 | -0.13 |
|  | 0.48 | 0.48 | 0.00 |
|  | 0.50 | 0.41 | -0.10 |
|  | 0.44 | 0.20 | -0.24 |
|  | 0.45 | 0.40 | -0.04 |
|  | 0.43 | 0.41 | -0.02 |
|  | 0.48 | 0.41 | -0.07 |
|  | 0.56 | 0.52 | -0.04 |
